# Age-dependent loss of cohesion protection in human oocytes

**DOI:** 10.1016/j.cub.2023.11.061

**Published:** 2023-12-21

**Authors:** Bettina P. Mihalas, Gerard H. Pieper, Mansour Aboelenain, Lucy Munro, Vlastimil Srsen, Cerys E. Currie, David A. Kelly, Geraldine M. Hartshorne, Evelyn E. Telfer, Andrew D. McAinsh, Richard A. Anderson, Adele L. Marston

**Affiliations:** 1 The Wellcome Centre for Cell Biology, Institute of Cell Biology, School of Biological Sciences, University of Edinburgh, Edinburgh EH9 3BF, UK; 2 Theriogenology department, Faculty of Veterinary Medicine, Mansoura University, Mansoura 35516, Egypt; 3 Institute of Cell Biology, School of Biological Sciences, University of Edinburgh, Edinburgh EH8 9XD, UK; 4 Centre for Mechanochemical Cell Biology & Division of Biomedical Sciences, Warwick Medical School, University of Warwick, Gibbet Hill, Coventry CV4 7AL, UK; 5 University Hospitals Coventry and Warwickshire NHS Trust, Coventry CV2 2DX, UK; 6 Medical Research Council Centre for Reproductive Health, University of Edinburgh, Edinburgh EH16 4TJ, UK

## Abstract

Aneuploid human eggs (oocytes) are a major cause of infertility, miscarriage, and chromosomal disorders. Such aneuploidies increase greatly as women age, with defective linkages between sister chromatids (cohesion) in meiosis as a common cause. We found that loss of a specific pool of the cohesin protector protein, shugoshin 2 (SGO2), may contribute to this phenomenon. Our data indicate that SGO2 preserves sister chromatid cohesion in meiosis by protecting a “cohesin bridge” between sister chromatids. In human oocytes, SGO2 localizes to both sub-centromere cups and the pericentromeric bridge, which spans the sister chromatid junction. SGO2 normally colocalizes with cohesin; however, in meiosis II oocytes from older women, SGO2 is frequently lost from the pericentromeric bridge and sister chromatid cohesion is weakened. MPS1 and BUB1 kinase activities maintain SGO2 at sub-centromeres and the pericentromeric bridge. Removal of SGO2 throughout meiosis I by MPS1 inhibition reduces cohesion protection, increasing the incidence of single chromatids at meiosis II. Therefore, SGO2 deficiency in human oocytes can exacerbate the effects of maternal age by rendering residual cohesin at pericentromeres vulnerable to loss in anaphase I. Our data show that impaired SGO2 localization weakens cohesion integrity and may contribute to the increased incidence of aneuploidy observed in human oocytes with advanced maternal age.

## Graphical abstract

**Graphical abstract:**
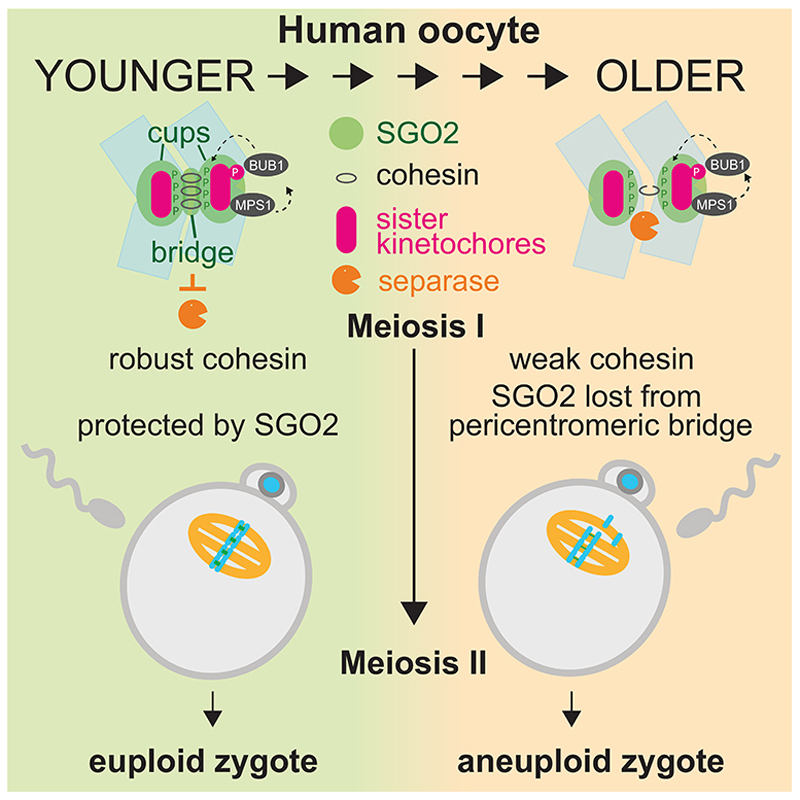

## Introduction

In humans, infertility and miscarriage are common, and a leading cause is aneuploidy in the egg (oocyte).^1^ Even among women at peak fertile age, over 20% of oocytes are aneuploid, whereas at advanced maternal age (>32 years), aneuploidy affects more than 50% of oocytes.^2^ This aneuploidy arises predominantly from errors in meiosis, the specialized cell division that generates oocytes that have half the number of chromosomes of the parental cell. Meiosis I segregates the homologous chromosomes, whereas meiosis II segregates the sister chromatids.^3^ Accurate sequential execution of meiosis I and II requires the establishment of cohesin complexes during DNA replication to link sister chromatids together, and the preservation of this cohesin must be maintained until chromosomes are ready to segregate. In mammalian oocytes, cohesin is established *in utero* after which oocytes enter a long arrest in meiotic prophase I. Meiosis is not resumed until the oocyte is ovulated, potentially several decades later. Evidence from mice indicates that there is no cohesin turnover during this period,^4,5^ suggesting that cohesin laid down in the fetus must last throughout the female reproductive lifespan, up to age ∼50 years in humans. However, female fertility declines before this, largely due to the loss of oocyte euploidy. Reduced sister chromatid cohesion is a key driver of aneuploidy in human oocytes, particularly with increased maternal age.^2,6–9^ Therefore, the molecular origin of the age-dependent cohesin loss in human oocytes requires investigation.

In most organisms, meiotic sister chromatid cohesion is conferred by a meiosis-specific variant of cohesin containing the REC8 kleisin, of which there are three spatially distinct populations: arm, pericentromeric, and centromeric cohesin.^10–13^ In meiosis I, separase-dependent cleavage of chromosomal arm REC8, which requires its prior phosphorylation, triggers homologous chromosome segregation.^14–21^ Centromeric cohesin joins sister kinetochores to ensure sister chromatid co-segregation in meiosis I, but its separase-dependent loss in late meiosis I individualizes sister kinetochores to allow their biorientation in meiosis II.^12,13^ Crucially, however, pericentromeric REC8 is protected from phosphorylation, and therefore cleavage in meiosis I, by shugoshin family proteins which recruit protein phosphatase 2A (PP2A) to the pericentromeres.^22–24^ Retention of pericentromeric cohesion in meiosis I permits sister chromatid alignment in meiosis II, which is followed by separase re-activation, cohesin de-protection, and sister chromatid segregation.^12,13,25^ In mouse oocytes, shugoshin 2 (SGO2) protects REC8 from separase: *SGO2* knockout mice are viable but infertile because all cohesin is cleaved in anaphase I, resulting in random segregation of sister chromatids at meiosis II.^24,26,27^ Consistent with its role in pericentromeric cohesin protection, mouse SGO2 localizes proximal to centromeres and spanning the junction between sister centromeres.^28^ This sister chromatid junction corresponds to the so-called inner centromere where both pericentromeric heterochromatin and protected cohesin reside. The pericentromeric localization of shugoshins 1 and 2 in both meiosis and mitosis is under control of the BUB1 and MPS1 kinases (reviewed in Marston and Wassmann^29^). In cultured human mitotic cells, MPS1 phosphorylates the kinetochore protein KNL1 to recruit BUB1-BUB3.^30,31^ BUB1, in turn, phosphorylates histone H2A on Thr120 to provide a docking site for shugoshin 1 (SGO1) and thereby protect mitotic cohesin from release via the separase-independent prophase pathway.^32,33^ However, in mouse oocytes, although BUB1 kinase activity promotes SGO2 localization to pericentromeres in meiosis I, it appears dispensable for fertility.^28,34^ Instead, pericentromeric cohesin protection in mouse oocytes requires MPS1, which, counterintuitively, promotes SGO2 localization predominantly to centromeres in meiosis I.^28^ Furthermore, mouse SGO2 delays separase-dependent cleavage of the centromeric pool of cohesin until late telophase I, thereby maintaining kinetochore monoorientation as homologous chromosomes segregate.^12^ Therefore, mouse SGO2 is a central regulator of the functionally distinct pools of cohesin in centromeres and pericentromeres, although it is unclear how this spatial and temporal resolution is achieved.

Cohesin-deficient mouse oocytes show aneuploidies similar to the age-related decline in human oocytes.^35^ Reduced levels of chromosomal cohesin and SGO2 with increased maternal age have been observed in some mouse strains^36–38^; however, female mice do not show reproductive aging to the same extent as humans. Therefore, understanding the role of cohesin protection in counteracting age-related aneuploidy requires direct analysis of human oocytes. The discovery of a frameshift mutation in *SGO2* as the likely cause of ovarian insufficiency and infertility in a female patient^39^ implies a critical role for SGO2 in human oogenesis, but the effects of aging on SGO2 and cohesin protection have not been analyzed. Here, we use super-resolution microscopy to show that in human metaphase I and II oocytes, the two sister kinetochores are embedded within SGO2 cups that connect through a pericentromeric bridge spanning the sister chromatid junction. Oocytes from older women frequently show a specific reduction of SGO2 at the bridge. Furthermore, inhibition of MPS1 in oocytes from younger women causes removal of SGO2 and loss of cohesion protection, recapitulating the phenotype of older oocytes. These findings support a model where age-dependent decline in association of SGO2 with the pericentromere bridge makes pericentromeric cohesion vulnerable to premature loss, a major cause of age-related aneuploidy in humans. It follows that the development of methods to preserve SGO2 may offer the potential to support fertility while reducing oocyte aneuploidy in women of older reproductive age.

## Results

### SGO2 cups and bridges sister kinetochores in human oocytes

To understand whether SGO2 could protect cohesin during the long prophase I arrest and meiotic divisions, we examined its localization in whole human oocytes at different meiotic stages (Figure S1A). SGO2 signal was detected in the germinal vesicle nucleus already during prophase I but became enriched on chromosomes only following germinal vesicle breakdown (GVBD), marking the end of prophase I arrest. By prometaphase I, SGO2 was focused around the inner kinetochore marker, CENP-C. SGO2 was also localized near the kinetochore marker in metaphase I and in metaphase II, at which point oocytes naturally arrest awaiting fertilization.

**Figure 1:**
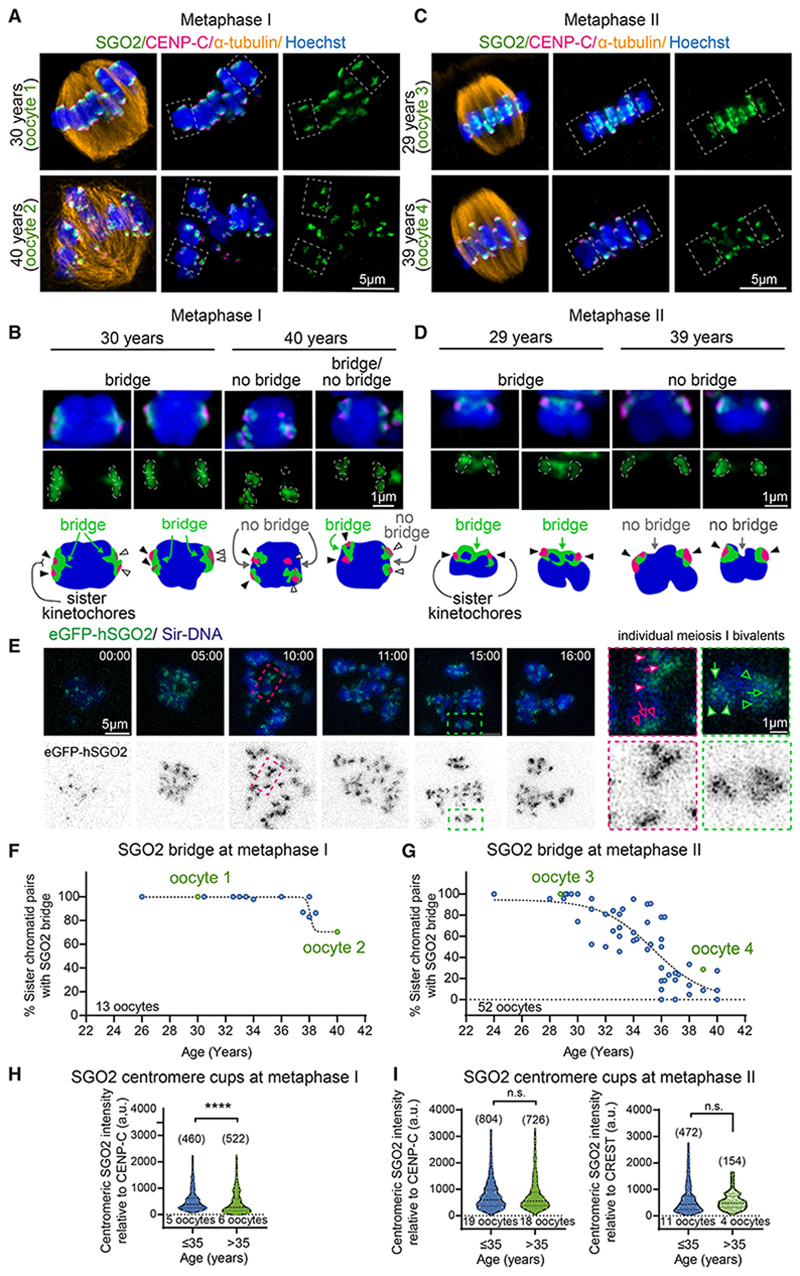
Age-dependent loss of human SGO2 from the pericentromeric bridge, but not centromeres, in meiosis I and II oocytes (A–D) Human SGO2 localizes to centromeric cups and an inter-sister pericentromere bridge in metaphase I oocytes and metaphase II oocytes of younger women but is lost from the bridge with age. Representative images of human metaphase I (A) and (B) and metaphase II oocytes (C) and (D) from younger (age 30 and 29 years) and older women (age 40 and 39 years). SGO2 (green), inner kinetochores (CENP-C, magenta), microtubules (α-tubulin, orange), and chromosomes (Hoechst, blue) are shown. White dashed line boxes indicate chromosomes further magnified in (B) metaphase I and (D) metaphase II. Centromeric localization of SGO2 is bounded by dashed lines. Interpretations of localizations observed are indicated in the schematics below. White and black arrowheads indicate pairs of sister centromeres, arrows represent the pericentromere where the SGO2 bridge is expected to be located. (E) Live imaging showing the localization of exogenous eGFP-SGO2 in human oocytes. Representative image showing eGFP-SGO2 (green) and sir-DNA (blue) at different time points (hh:mm) of meiosis I. Magenta and green dashed line boxes indicate DNA and eGFP-SGO2 further magnified on right. Arrowheads indicate eGFP-SGO2 cups at centromeres, and arrows indicate eGFP-SGO2 at the bridge. Pairs of sister centromeres are indicated with either black or white filled arrows. n = 5 microinjected oocytes. (F and G) The percentage of chromatids per oocyte with SGO2 localization at the pericentromeric bridge was scored relative to the woman’s age at MI (F) and MII (G). Data were fit to a sigmoidal, 4 parameter logistic curve (metaphase I; R^2^ = 0.84, metaphase II; R^2^ = 0.67). Oocytes shown in (A)–(D) are labeled (green circles). (H and I) The relative intensity of the centromeric pool of SGO2 in (H) metaphase I and (I) metaphase II oocytes from younger (age ≤ 35 years) or older women (age ≤ 35 years) was measured in arbitrary units (a.u.) relative to the inner kinetochore (CENP-C) or centromere (CREST) markers. Plots show median (dashed black line) and 25th and 75th percentiles (dotted black lines). p values were calculated using the Mann-Whitney test: CENP-C (p = 0.37) and CREST (p = 0.15) ****p < 0.0001; n.s., not significant. See also Figure S1 and Videos S1 and S2.

To observe SGO2 localization in more detail, we used super-resolution microscopy on whole human oocytes at meta-phase I to obtain 3D reconstructions and analyzed individual bivalents (homologous chromosomes connected by chiasmata). Human SGO2 surrounded centromeres and spanned the junction between sister centromeres, which we refer to as the pericentromeric bridge (Figures 1A and 1B; age 30 years). Centromeric SGO2 surrounds, but does not overlap, the two sister centromeres/inner kinetochores (as identified by calcinosis, Raynaud phenomenon, esophageal dysmotility, sclerodactyly, telangiectasia [CREST] or CENP-C staining), forming cup-like structures that are frequently connected by a SGO2 bridge (Figure 1B). In metaphase II, where sister kinetochores form a back-to-back configuration, the SGO2 bridge was found to span the distance between the SGO2 centromeric cups (Figures 1C and 1D; age 29 years). Line scans of metaphase II chromosomes further suggested the existence of separate centromeric and bridge SGO2 pools (Figure S1B). We additionally examined ectopic SGO2-GFP localization by live cell imaging of human meiosis I and bovine meiosis I and II oocytes after injection of *eGFP-SGO2* mRNA, which corroborated the cup and bridge structure observed in fixed human oocytes (Figures 1E and S1D; Videos S1 and S2). Therefore, human SGO2 is positioned at sub-centromere cups and a pericentromeric chromatin bridge during the oocyte meiotic divisions, consistent with a role in pericentromeric cohesin protection.

### Age-dependent loss of SGO2

Although SGO2 centromeric cups were consistently observed, a SGO2 bridge was not always present (Figures 1A–1D; age 40 and 39 years), and line scans confirmed the specific absence of the SGO2 pool at the pericentromeric bridge on some chromosomes (Figure S1B). Scoring the frequency of sister chromatid pairs with an intact SGO2 bridge in meiosis I (Figure 1F) and II (Figure 1G) revealed a relationship between the presence of the SGO2 bridge and the woman’s age. In oocytes from women aged 30 years and under, a SGO2 bridge was observed at both metaphase I and metaphase II on almost all sister chromatid pairs (Figures 1F and 1G). At higher maternal ages, however, the frequency of sister chromatids with a SGO2 bridge was decreased. This effect was most obvious in meiosis II oocytes, where the back-to-back configuration of sister kinetochores facilitates observation of the pericentromeric region. Accordingly, at over 30 years of age, the frequency of chromosomes lacking a SGO2 bridge in meiosis II increased, and oocytes from most women aged 36 years or over lacked an SGO2 bridge on the majority of sister chromatid pairs (Figure 1G). Although the number of oocytes was small and it is challenging to distinguish centromeric and bridge SGO2 when kinetochores are in the side-by-side orientation, the data hint that bridge SGO2 was already depleted in meiosis I with maternal age (Figure 1F). The age dependence of the SGO2 bridge was also not affected by whether the oocytes had been acquired during *in vitro* fertilization (IVF) or intracytoplasmic sperm injection (ICSI) treatment (Figure S1C). Measurement of centromeric SGO2 signal (normalized to CREST or CENP-C) revealed a small, but significant, decrease with age at metaphase I, whereas there was no significant age-dependent difference in centromeric SGO2 at meta-phase II (Figures 1H and 1I). We conclude that in human oocytes at metaphase I and II, SGO2 exists in two chromosomal pools, the cups and the bridge, and that the pool of SGO2 at the pericentromeric bridge, where protected cohesin resides, is particularly vulnerable to aging.

### Absence of the SGO2 bridge is associated with increased inter-sister kinetochore distance

**Figure 2:**
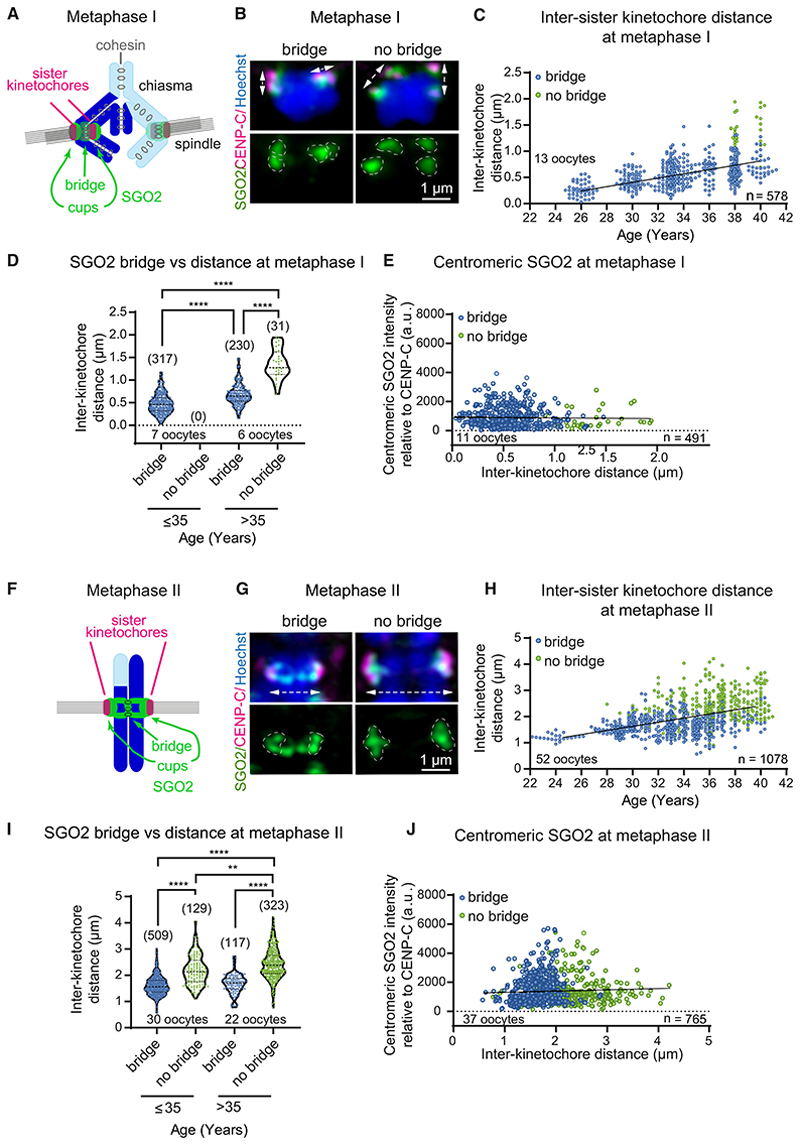
Loss of SGO2 at the pericentromeric bridge with increased inter-sister kinetochore distance (A) Schematic summarizing SGO2 localization in metaphase I oocytes from younger women. (B) Inter-sister kinetochore distance (white dashed arrows) was determined on metaphase I chromosomes and related to the presence of the SGO2 bridge. (C) Increase in inter-sister kinetochore distance at metaphase I with female age for chromosomes with a SGO2 bridge (blue) or no bridge (green). Data were fit to a linear regression (R^2^ = 0.81; p < 0.0001). (D) Inter-sister kinetochore distance for sister chromatid pairs with a SGO2 bridge or no bridge at metaphase I in younger (age ≤ 35 years) or older women (age >35 years). Plots show median (dashed black line) and 25th and 75th percentiles (dotted black lines). Statistical analyses were performed using the Kruskal-Wallis test (****p < 0.0001). (E) Centromeric SGO2 does not correlate with inter-sister kinetochore distance at MI. The relative intensity of the centromeric pool of SGO2 metaphase I oocytes was measured in arbitrary units (a.u.) relative to the kinetochore marker CENP-C (p = 0.576; R^2^ = 0.0006). Data were fit to a linear regression. (F) Schematic summarizing SGO2 localization in metaphase II oocytes from younger women. (G) Inter-sister kinetochore distance (white dashed arrows) was determined on metaphase II chromosomes and related to the presence of the SGO2 bridge. (H) Increase in inter-sister kinetochore distance at metaphase II with female age for sister chromatid pairs with a SGO2 bridge (blue) or no bridge (green). Data were fit to a linear regression (R^2^ = 0.47; p < 0.0001). (I) Inter-sister kinetochore distance where the SGO2 bridge is present or absent at metaphase II in younger (age ≤ 35 years) or older women (age >35 years). Statistical analyses were performed using the Kruskal-Wallis test (****p < 0.0001, **p = 0.0017). Plots show median (dashed black line) and 25th and 75th percentiles (dotted black lines). (J) Centromeric SGO2 does not correlate with inter-sister kinetochore distance at metaphase II. The relative intensity of the centromeric pool of SGO2 metaphase II oocytes was measured in arbitrary units (a.u.) relative to the kinetochore marker CENP-C (p = 0.23; R^2^ = 0.0019). Data were fit to a linear regression. See also Figure S2.

Increased inter-sister kinetochore distance, indicative of a loss of centromeric cohesion, has been observed in aged human oocytes.^7–9^ To understand the relationship between cohesion loss and the lack of a SGO2 bridge, we measured the inter-sister kinetochore distance at metaphase I and II and correlated this to the presence or absence of SGO2 staining at the bridge (Figures 2A–2J; note that in metaphase I, we defined the bridge as continuous SGO2 staining between the CENP-C/Crest centromere signals). As expected, we observed a clear correlation between sister kinetochore distance and female age at both metaphase I and metaphase II (Figures 2C and 2H), which was consistent with whether the women had undergone IVF or ICSI treatment (Figure S2A), confirming that centromeric cohesion weakens with age. Additionally, meiosis I and II sister kinetochores that lack a SGO2 bridge tended to be further apart (Figures 2C, 2D, 2H, and 2I). Although sister kinetochore pairs with a SGO2 bridge showed increased separation in meiosis I oocytes from older (age >35 years) compared with younger (age ≤ 35 years) women, the greatest inter-sister kinetochore distance was observed on those pairs lacking a SGO2 bridge (Figure 2D). Similarly, even in metaphase II oocytes from young (age ≤ 35 years) women, sister chromatid pairs lacking a SGO2 bridge were further apart than pairs with a SGO2 bridge (Figure 2I). In contrast, centromeric SGO2 signal did not correlate with inter-sister kinetochore distance at either metaphase I or metaphase II (Figures 2E, 2J, and S2B). Therefore, regardless of oocyte age, decreased centromeric cohesion is associated with specific loss of the bridge, but not centromeric, SGO2.

### Incidence of single chromatids in meiosis II oocytes is higher where many sister chromatid pairs lack a SGO2 bridge

**Figure 3:**
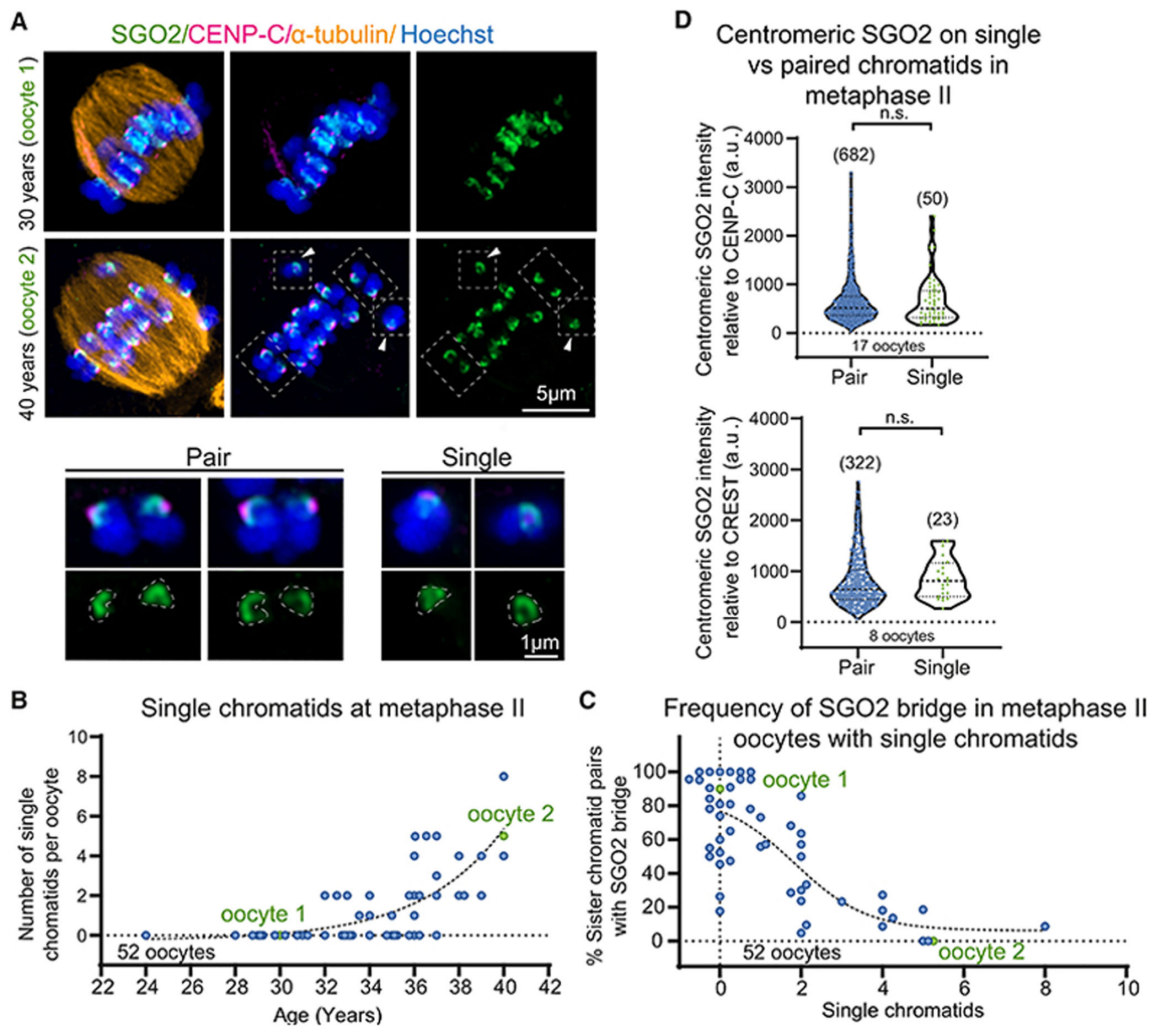
Loss of cohesion in metaphase II oocytes with age and absence of bridge SGO2 (A) Representative image showing presence of single chromatids (arrows) in human metaphase II oocytes from an older (age 40) woman, compared with a younger (age 30) woman without single chromatids. White boxes with dashed lines indicate examples of paired and single chromosomes in oocyte 2. SGO2 (green), inner kinetochores (CENP-C; magenta), microtubules (α-tubulin; orange), and chromosomes (Hoechst; blue) are shown. (B) Increase of single chromatids in metaphase II oocytes with maternal age. The number of single chromatids was scored relative to woman’s age. Data were fit to a sigmoidal, 4 parameter logistic curve (R^2^ = 0.57). Oocytes used in representative images are labeled in the graphs. (C) Increased number of single chromatids in metaphase II oocytes with an increased fraction of sister chromatid pairs lacking a SGO2 bridge. Data were fit to a sigmoidal, 4 parameter logistic curve (R^2^ = 0.60). (D) The relative intensity of the centromeric pool of SGO2 between paired and single chromatids was measured only from oocytes that had single chromatids. SGO2 intensity was measured in arbitrary units (a.u.) relative to the kinetochore markers CENP-C (p = 0.96, Mann-Whitney test) and CREST (p = 0.24, Mann-Whitney test). Plots show median (dashed black line) and 25th and 75th percentiles (dotted black lines). p values were calculated using the Mann-Whitney test. n.s., not significant. See also Figure S3.

In the most extreme, defective centromeric cohesion would abolish all linkages between sister chromatids, resulting in the presence of single chromatids in naturally arrested metaphase II oocytes. To further understand the consequences of defective sister chromatid cohesion in aged human oocytes (Figures 2C and 2H), we scored the number of single chromatids in our 3D metaphase II reconstructions (Figure 3A). As expected,^7^ this revealed a sharp increase in single chromatids within oocytes from women >35 years of age, independent of their treatment (Figures 3B and S3A). More single chromatids were observed in oocytes with an elevated frequency of sister chromatid pairs lacking a SGO2 bridge (Figure 3C). In contrast, centromeric SGO2 signal was comparable on single and paired sister chromatids (Figure 3D), indicating that association of SGO2 with centromeric cups is independent of cohesion. Single chromatids were rarely detected in meiosis I oocytes from women of any age (Figure S3B). This indicates that chromosome arms have sufficient cohesion to ensure chromatids remain juxtaposed and that it is only after loss of arm cohesion, when sister chromatids rely solely on centromeric cohesion, that the consequences of defective centromeric cohesion are apparent.

### SGO2 colocalizes with PP2A and REC8 at pericentromeres in meiosis II

**Figure 4:**
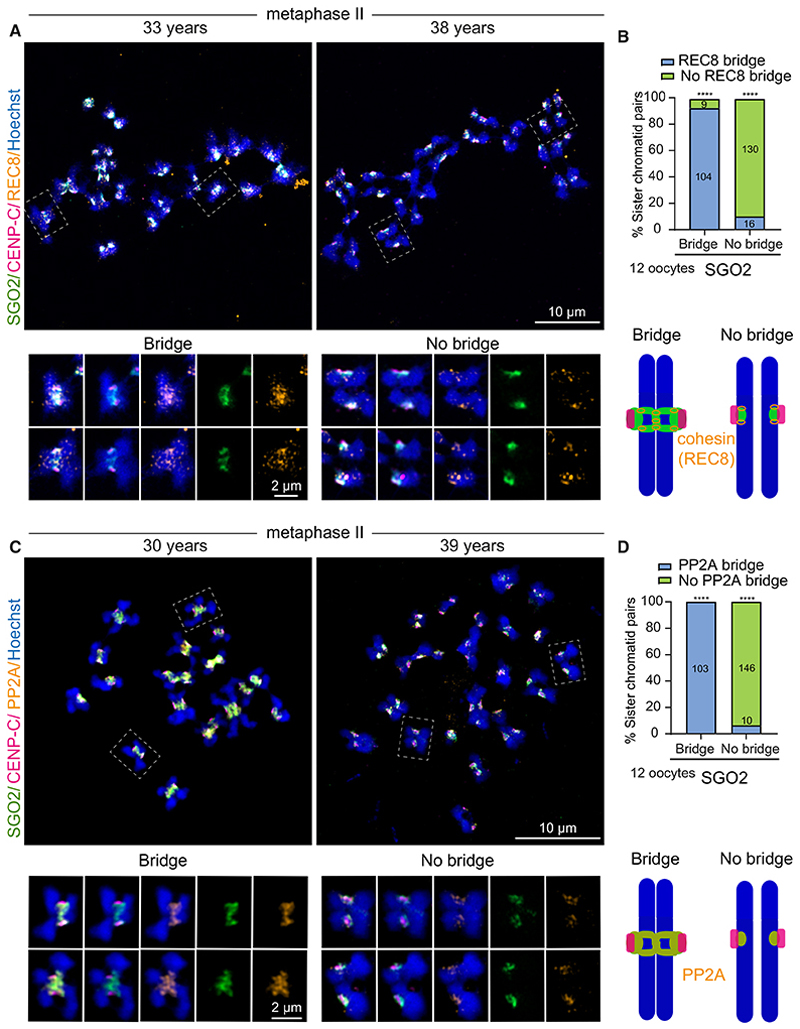
Co-localization of PP2A and cohesin with the SGO2 pericentromere bridge on human metaphase II chromosomes (A and B) Chromosome spreads of metaphase II-arrested human oocytes were stained with antibodies against SGO2 (green), inner kinetochores (CENP-C, magenta), and cohesin (REC8, orange). Chromosomes were stained with Hoechst (blue). (A) Representative images with white dashed line boxes indicating representative chromosome figures shown at higher magnification below. (B) Localization of REC8 at the pericentromere bridge was scored for sister chromatid pairs with and without a SGO2 bridge. Schematic representations of the data are shown below. Statistical analyses were performed using the chi-squared test (****p < 0.0001). (C and D) Chromosome spreads of metaphase II-arrested human oocytes were stained with antibodies against SGO2 (green), inner kinetochores (CENP-C, magenta), and PP2A (orange). Chromosomes were stained with Hoechst (blue). (C) Representative images with white dashed line boxes indicating representative chromosome figures shown at higher magnification below. (D) Localization of PP2A at the pericentromere bridge was scored for sister chromatid pairs with and without a SGO2 bridge. Statistical analyses were performed using the chi-squared test (****p < 0.0001). Schematic representations of the data are shown below. See also Figure S4.

In yeast and mouse, it is well-established that shugoshins protect REC8-cohesin until anaphase II of meiosis through recruitment of PP2A to pericentromeric regions.^22–24,40^ Our findings above revealed a relationship between centromeric cohesion and the SGO2 bridge, suggesting that this pool of SGO2 might be most relevant in REC8 protection or that SGO2 might rely on robust pericentromeric cohesion for its bridge localization. To test these ideas further, we compared SGO2 and REC8 localization on spread metaphase II chromosomes from human oocytes. At this stage, we expected arm cohesin to have been lost so that only centromeric cohesin remains. Consistently, REC8 was detected only in centromeric and pericentromeric regions (Figure 4A). We confirmed that the REC8 antibody was specific, since no signal was detected in zygotic pronuclei or de-condensed sperm (Figures S4A and S4B). As in whole oocytes, SGO2 on metaphase II spreads encircled centromeres (as identified by CENP-C staining) and frequently forms a pericentromeric bridge across the sister centromere junction (Figure 4A). Importantly, the SGO2 signal partially overlapped with REC8, and there was a strong correlation between the presence of a SGO2 bridge and REC8 across the sister chromatid junction (Figure 4B). Similarly, PP2A co-localized with SGO2 at the pericentromere bridge on metaphase II chromosome spreads but was absent from the pericentromere on chromosomes that lacked a SGO2 bridge (Figures 4C and 4D). Although the majority of donated human oocytes were at the metaphase II stage, we were able to analyze a small number of metaphase I chromosome spreads (Figures S4C and S4D), where line scans confirmed the presence and absence of a SGO2 bridge even within the same bivalent (Figures S4E and S4F). We also found that SGO2 and PP2A signal co-localized on metaphase I spreads and correlated in intensity within an oocyte (Figures S4C–S4I). These findings reveal co-localization of SGO2 and PP2A at the centromere cups and the pericentromeric bridge in human metaphase I and II oocytes. They also show that SGO2-PP2A colocalizes with the protected pool of REC8 spanning the sister chromatid junction in metaphase II.

### SGO2 localization requires MPS1 and BUB1 activity

**Figure 5:**
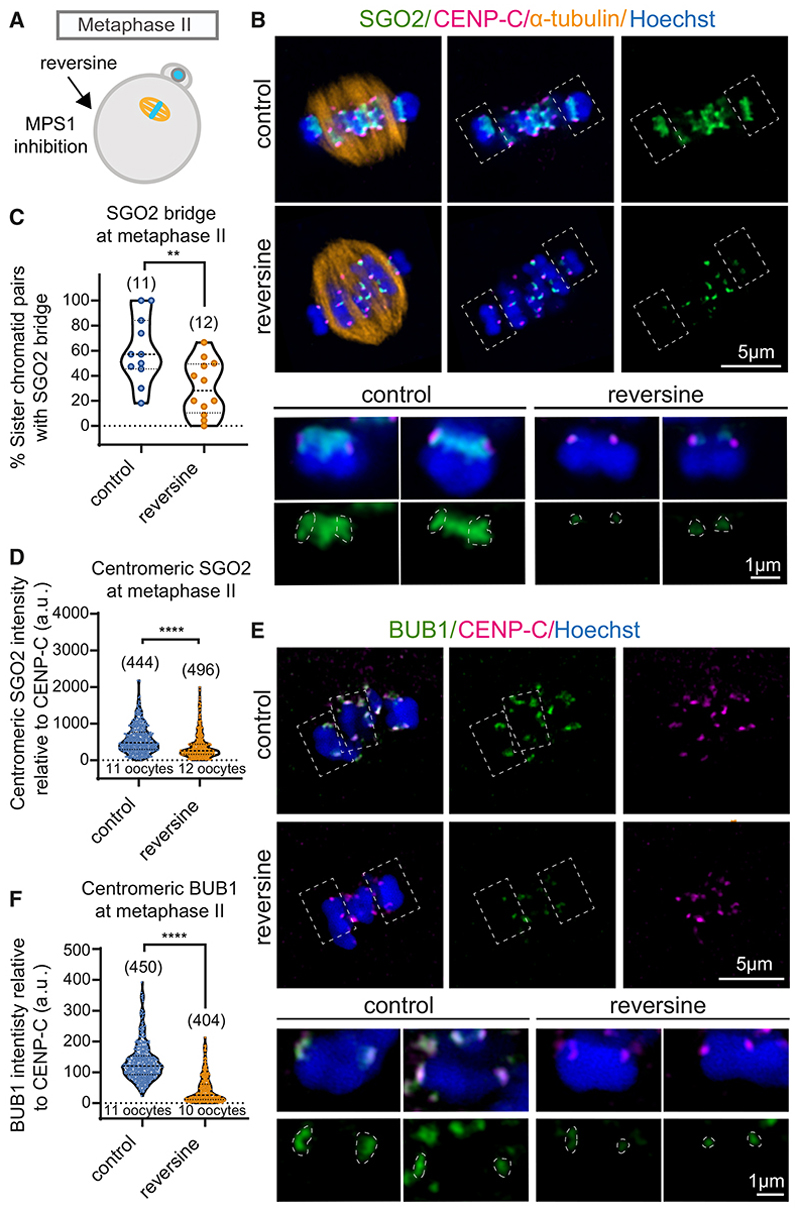
SGO2 localization to the bridge and centromere requires MPS1 activity (A–D) Inhibition of MPS1 in metaphase II oocytes impairs SGO2 localization. (A) Scheme of the experiment. Metaphase II eggs from women aged ≤ 36 years were treated with 500 nM reversine^44^ (to inhibit MPS1) or DMSO (control) overnight and fixed. (B) Representative images of control and reversine-treated metaphase II oocytes after immunostaining with antibodies against SGO2 (green), CENP-C (inner kinetochores, magenta), and α-tubulin (microtubules, orange). White boxes with dashed lines indicate chromosomes that have been further magnified below. (C) The percentage of chromatids per oocyte with SGO2 localization at the pericentromeric bridge was scored in control and reversine-treated meta-phase II oocytes (**p = 0.0084; Welch’s t test). (D) The relative intensity of the centromeric pool of SGO2 in metaphase II oocytes relative to CENP-C in control and reversine-treated oocytes (****p < 0.0001; Mann-Whitney test). (E and F) MPS1 activity is required for BUB1 localization to kinetochores. Metaphase II oocytes treated with reversine as in (A) were immunostained with antibodies against BUB1 (green), CENP-C (inner kinetochore; magenta), and counter-stained with Hoechst (blue) to visualize chromosomes. (E) Representative images of BUB1 localization in control and reversine-treated metaphase II oocytes. White boxes with dashed lines represent chromosomes that have been further magnified below. White dashed circles show examples of area selections for BUB1 intensity measurements. (F) The relative intensity of the centromeric BUB1 in metaphase II oocytes from control and reversine-treated oocytes relative to CENP-C (****p < 0.0001; Mann-Whitney test). Plots show median (dashed black line) and 25th and 75th percentiles (dotted black lines). See also Figure S5.

**Figure 6:**
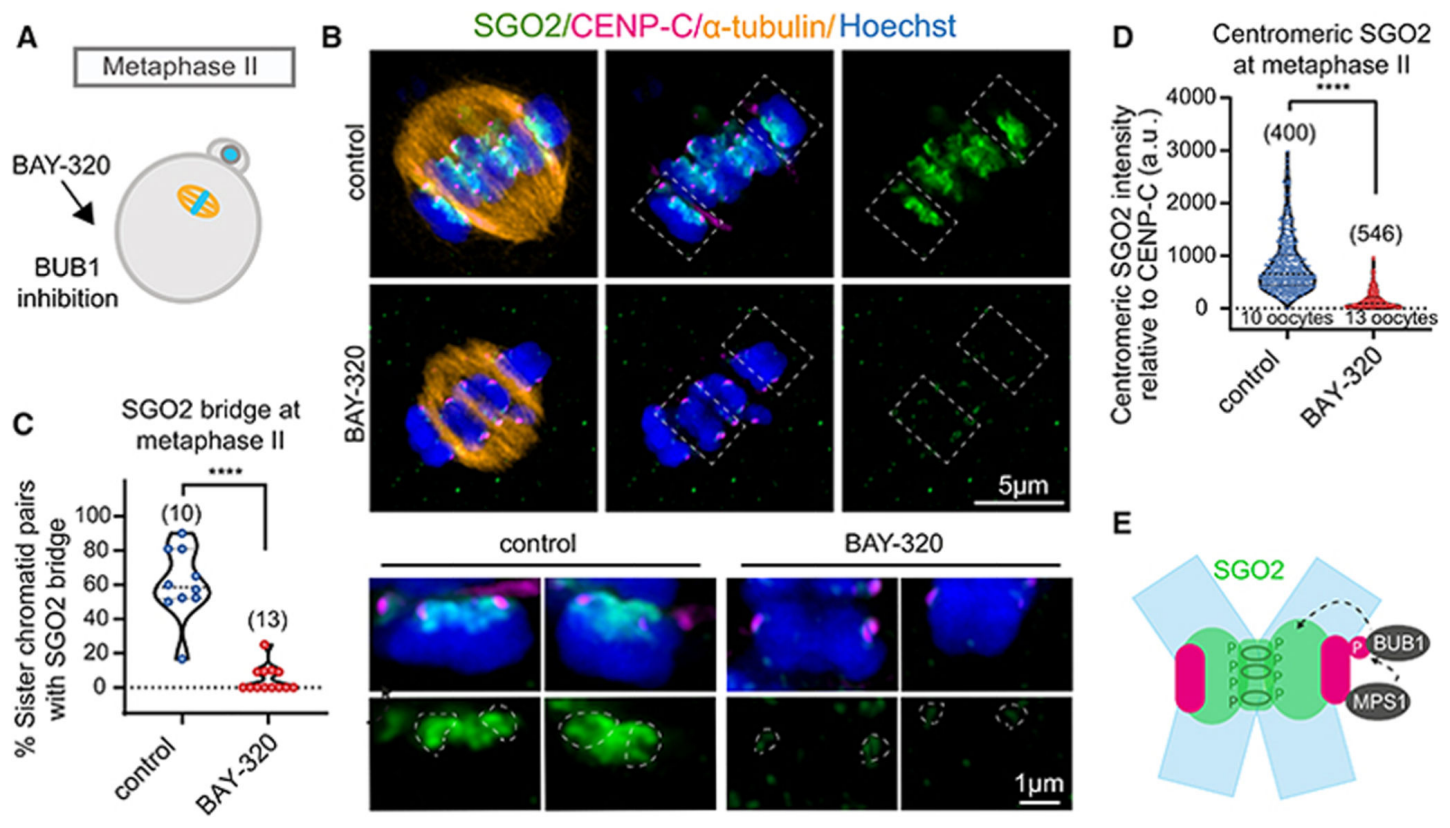
Dependence of SGO2 localization on BUB1 activity (A) Scheme of the experiment. Metaphase II oocytes from women aged ≤ 36 years were treated with 10 μM BAY-320 (to inhibit BUB1^47^) or DMSO (control) overnight and fixed. (B) Representative images of control and Bay-320-treated metaphase II oocytes after immunostaining with antibodies against SGO2 (green), CENP-C (inner kinetochores, magenta), and α-tubulin (microtubules, orange). White boxes with dashed lines indicate chromosomes that have been further magnified below. (C) The percentage of chromatids per oocyte with SGO2 localization at the pericentromeric bridge was scored in control and BAY-320-treated metaphase II oocytes (****p < 0.0001, Mann-Whitney test). (D) The relative intensity of the centromeric pool of SGO2 in metaphase II oocytes from control and BAY-320-treated oocytes were measured relative to CENP-C. (****p < 0.0001, Mann-Whitney test.) Plots show median (dashed black line) and 25th and 75th percentiles (dotted black lines). (E) Schematic summarizing a model for MPS1 and BUB1 in SGO2 localization in metaphase II oocytes from younger women. See also Figure S6.

In human mitotic cells and mouse oocytes, MPS1 and BUB1 kinase activities promote the localization of shugoshin proteins to pericentromeres.^28,32,33,41–43^ To understand the role of these kinases in localizing human SGO2 to the centromere and bridge in oocytes, we used specific inhibitors. Human metaphase II oocytes were incubated with the MPS1 inhibitor, reversine,^44^ for 16 h before fixing and staining by immunofluorescence (Figures 5A and 5B). MPS1 inhibition in metaphase II oocytes resulted in loss of the SGO2 bridge, together with a significant reduction in centromeric SGO2 signal (Figures 5B–5D). MPS1 is required for chromosome biorientation and error correction in mitotic cells, and its inhibition is predicted to increase the attachment of sister kinetochores to microtubules from the same pole.^30^ Accordingly, we observed a reduction in interkinetochore distance upon MPS1 inhibition in metaphase II cells, although the number of single chromatids was not increased (Figure S5). MPS1 activity localizes BUB1 to kinetochores in human mitotic cells^45,46^ and oocytes.^7^ Consistent with this, we found that the intensity of centromere-proximal BUB1 signal was greatly reduced after treating metaphase II oocytes with reversine (Figures 5E and 5F). We therefore tested the role of BUB1 in SGO2 localization by treatment with the BUB1 inhibitor Bay-320^47^ (Figure 6A). BUB1 inhibition in metaphase II oocytes resulted in almost complete loss of the SGO2 signal at both the centromeric cups and bridge (Figures 6B–6D), although this was not accompanied by an increase in either inter-sister kinetochore distance or the frequency of single chromatids (Figure S6). We conclude that the activities of both MPS1 and BUB1 contribute to localizing SGO2 at both the centromeric cups and inter-sister kinetochore bridge in human metaphase II oocytes and that the role of MPS1 may be executed through BUB1 recruitment (Figure 6E).

### SGO2 protects pericentromeric cohesion

**Figure 7:**
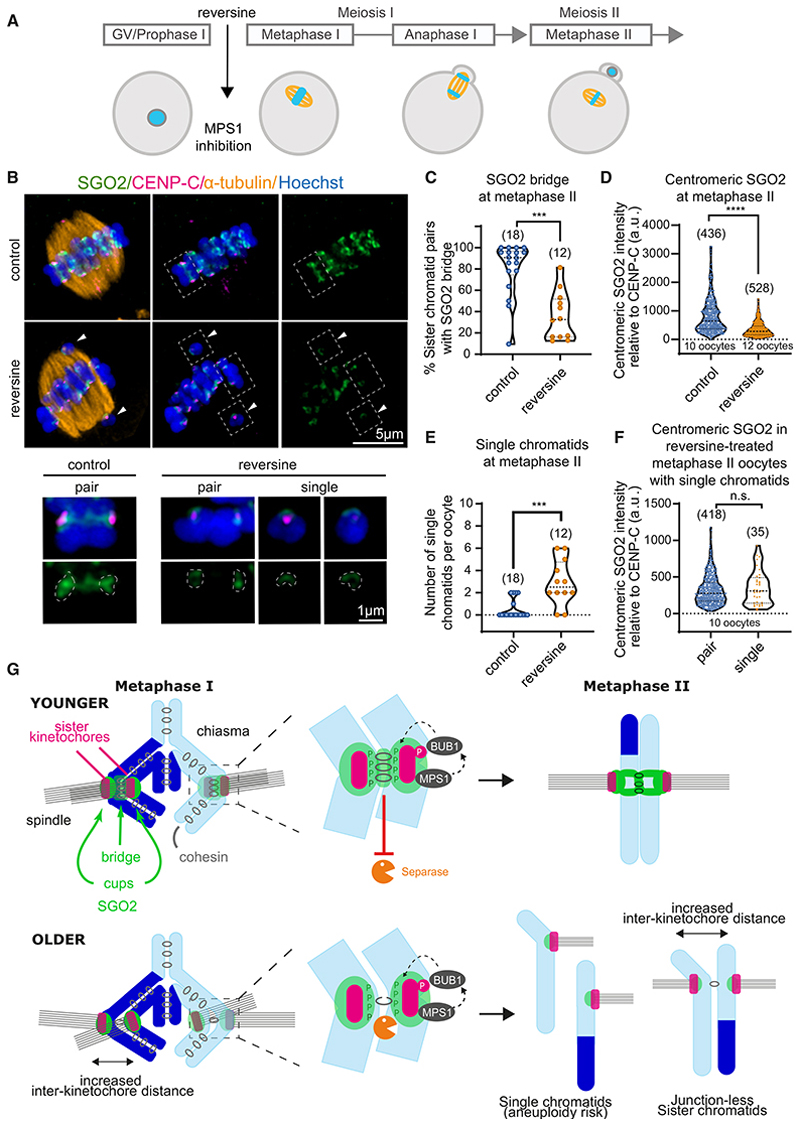
MPS1-dependent SGO2 protects pericentromeric cohesion during human meiosis (A) Schematic of experiment. Oocytes from women undergoing ICSI aged ≤ 36 years provided at or prior to GVBD were treated with 500 nM reversine or DMSO, allowed to mature to metaphase II for up to 24 h and fixed. (B) Representative images of oocytes treated with reversine from GVBD stage alongside control oocytes after immunostaining with antibodies against SGO2 (green), CENP-C (inner kinetochore, magenta), α-tubulin (microtubules, orange), and counter-staining with Hoechst (chromosomes, blue). White boxes with dashed lines represent chromosomes that have been further magnified below. (C) The percentage of chromatids per oocyte with SGO2 localization at the pericentromeric bridge was scored (***p = 0.0001; Mann-Whitney test). (D) The relative intensity of the centromeric pool of SGO2 relative to CENP-C. (****p < 0.0001; Mann-Whitney test). (E) The number of single chromatids observed in metaphase II oocytes after treatment with reversine from GVBD compared with controls (***p < 0.0003; Mann-Whitney test). (F) Centromeric SGO2 was measured in metaphase II oocytes that had single chromatids after reversine treatment from GVBD stage onward, and values for paired and single chromatids were compared. SGO2 intensity was measured in arbitrary units relative to CENP-C. Plots show median (dashed black line) and 25th and 75th percentiles (dotted black lines) (p = 0.57; Mann-Whitney test). n.s., not significant. (G) Model for age-dependent loss of cohesin protection. In oocytes from younger women, cohesion is maintained between sister chromatids. MPS1 at the kinetochore recruits BUB1, which phosphorylates histone H2A-Thr120 in the centromeric chromatin. This phosphorylation allows for the recruitment of SGO2 at the centromere and the pericentromeric bridge coincident with cohesin. During anaphase I, SGO2 protects cohesin within the pericentromeric bridge from separase activity to ensure that sister chromatids remain together at metaphase II and disjoin accurately only upon fertilization-triggered anaphase II. In oocytes from older women, pericentromeric cohesin deteriorates. This both increases inter-sister kinetochore distances and results in loss of SGO2 from the pericentromeric bridge. In the absence of SGO2 at the bridge, residual pericentromeric cohesin is vulnerable to separase-dependent cleavage in anaphase I. Consequently, sister chromatids risk premature separation and such single chromatids at metaphase II will disjoin randomly at fertilization resulting in increased incidences of aneuploid conceptions in older women. See also Figure S7.

We hypothesized that loss of SGO2 from the pericentromeric bridge in aged oocytes leaves residual centromeric cohesion vulnerable to cleavage by separase already in anaphase I. This predicts that artificial removal of SGO2 in meiosis I in younger oocytes would recapitulate the premature loss of centromeric cohesion observed in older oocytes (Figure 3B). To test this idea, we obtained oocytes at prophase I (containing a germinal vesicle nucleus) and matured them *in vitro*. Upon GVBD breakdown, either reversine or, as a control, DMSO was added, and oocytes were allowed to progress through the meiotic divisions before fixing at metaphase II (Figure 7A). Oocytes from women of 36 years or younger were selected for this experiment since their untreated oocytes are expected to retain SGO2 on the pericentromeric bridge (Figures 1F and 1G). Overall, around 81% (51/63) donated oocytes underwent GVBD *in vitro*, of which 75% (21/28) control and 65% (15/23) reversine-treated oocytes progressed to meiosis II (Figure S7B). As expected, MPS1 inhibition by addition of reversine after GVBD led to a loss of SGO2 signal from centromeres and the bridge at metaphase II, whereas control oocytes retained SGO2 in both locations (Figures 7B–7D). Chromosome alignment defects and reduced inter-sister kinetochore distance at metaphase II were also observed after MPS1 inhibition from GVBD/prophase I (Figure S7C). However, in contrast to oocytes treated with reversine only at metaphase II (Figure S5C), MPS1 inhibition from GVBD/prophase I led to a significant increase in single chromatids in metaphase II, indicating loss of centromeric cohesion already in meiosis I (Figure 7E). Therefore, inhibition of MPS1 from prior to metaphase I causes single chromatids in metaphase II, whereas inhibition only in metaphase II does not (Figure S5C). This demonstrates that MPS1 inhibition must abrogate the protection of pericentromeric cohesion in meiosis I, so that separase cleaves pericentromeric cohesin at the same time as arm cohesin in anaphase I. Taken together with the critical role of MPS1 in localizing SGO2 to pericentromeres (Figures 5B–5D and 7B–7D), these data strongly suggest that MPS1 protects pericentromeric cohesin in anaphase I through SGO2 recruitment prior to this stage. Centromeric SGO2 signal was comparable on pairs and single chromatids after MPS1 inhibition in meiosis I, implying that this SGO2 pool is insufficient to prevent premature cohesin loss (Figure 7F). Therefore, our findings implicate SGO2 at the pericentromeric bridge in protecting pericentromeric REC8 at the inter-sister kinetochore junction from cleavage during anaphase I to ensure that robust sister chromatid cohesion is retained at centromeres until metaphase II (Figure 7G). Given our finding that SGO2 at the bridge is lost in oocytes of older women, these results indicate that loss of cohesion protection could contribute to age-related aneuploidy.

## Discussion

A major limitation in the reproductive lifespan of women is the high rates of aneuploidy caused by meiotic errors in the oocyte,^1^ which contribute to increasing rates of infertility, miscarriage, and chromosomal disorders at older ages. A key driver of this aneuploidy is premature loss of centromeric cohesion,^2,7–9^ which prevents sister chromatids from aligning on the meiosis II spindle, leading to their mis-segregation in the second meiotic division and production of an aneuploid zygote upon fertilization. Here, we have provided evidence for age-dependent loss of the protector of pericentromeric cohesin as a potential mechanism predisposing sister chromatids to increased meiosis II mis-segregation in oocytes from older women.

### Loss of SGO2 from the pericentromeric bridge with age

Our super-resolution analysis of SGO2 localization in aged human oocytes revealed two pools of SGO2, corresponding to the centromeric cups and the pericentromeric bridge. We showed that in aged human oocytes, SGO2 is preferentially lost from the pericentromeric bridge, whereas it is mostly retained at centromeres since only a modest decrease was observed at metaphase I, but not metaphase II. The SGO2 pericentromeric bridge is located at the inter-sister kinetochore junction where REC8 is retained in metaphase II and therefore corresponds to the domain of protected pericentromeric cohesin. A reduction or absence of SGO2 in this region, as observed in aged oocytes, would therefore be expected to render pericentromeric cohesin unprotected and susceptible to cleavage by separase during anaphase I. This premature loss of sister chromatid cohesion is observed as single chromatids in metaphase II (Figure 7G).

What is the molecular cause for the absence of SGO2 at the pericentromeric bridge? A clue came from our finding that SGO2 loss from the pericentromeric bridge correlated with increased sister kinetochore distance even in oocytes from young women. In mitosis, SGO1, which protects centromeric cohesin not from separase-dependent cleavage but from non-proteolytic removal by the prophase pathway,^48^ is localized to the inner centromere by binding cohesin.^33^ It is not clear if SGO2 is similarly localized through binding cohesin in human oocytes, but these observations raise the possibility that loss of SGO2 from the bridge is a consequence of the deterioration of cohesin in aged oocytes. Consistent with this idea, although REC8 levels are reported to be reduced already at the GV stage in older women,^49^ we found that SGO2 associates with pericentromeres only after GVBD (Figure S1A). In this case, loss of bridge SGO2 would add a further vulnerability to aged oocytes by exposing the already critically low levels of pericentromeric cohesin to separase-dependent proteolysis. Single chromatids were only rarely observed in metaphase I (Figure S3B), indicating that even in aged oocytes, sufficient cohesin typically remains to hold sister chromatids together. It is only after separase activation at anaphase I that single chromatids are observed (Figure 3B). This indicates that either the age-dependent demise of cohesin leaves insufficient linkages at centromeres to sustain sister chromatid cohesion in the absence of arm cohesin or that loss of the SGO2 protector leads to premature cleavage of any centromeric cohesin that survived the age-dependent deterioration. Although these possibilities are not mutually exclusive, our demonstration of the importance of SGO2 in centromeric cohesin maintenance after metaphase I validates defective pericentromeric cohesion protection as a potential contributing factor to age-dependent aneuploidy. Removal of SGO2 in prophase I by inhibiting MPS1 led to loss of centromeric cohesion and single chromatids at metaphase II (Figure 7E). These observations also confirm that SGO2 is relevant for cohesin protection in human oocytes and provide a molecular explanation for infertility in a patient with a frameshift *SGO2* mutation.^39^

### MPS1 and BUB1-dependent SGO2 localization

We found that the kinase activities of both MPS1 and BUB1 are required for localization of SGO2 to the centromeric cups and pericentromeric bridge in human oocytes. Since MPS1 activity is required for BUB1 localization to kinetochores in human mitotic cells^30,31^ and oocytes^7^ (Figures 5E and 5F), the most straightforward explanation for these observations is that MPS1 promotes SGO2 recruitment through localizing BUB1. BUB1 is known to phosphorylate histone H2A on T120 in the centromere to provide a mark that is bound directly by Sgo1 in mitotic cells.^33,50^ If a similar mechanism operates in human oocytes, BUB1-dependent H2A-T120 phosphorylation could explain human SGO2 localization at the cups surrounding centromeres. In mouse oocytes, inhibition of MPS1 or BUB1 also reduces SGO2 localization at both centromeres and pericentromeres, with MPS1 inhibition primarily affecting centromeres and BUB1 primarily affecting pericentromeres. However, in mouse, MPS1, and not BUB1, is required for cohesin protection, suggesting that the protective pool of mSGO2 resides at centromeres.^28^ This idea is supported by the finding that artificial cleavage of centromeric cohesin in mouse meiosis I oocytes, while leaving pericentromeric cohesin largely intact, abolished cohesin protection.^12^ In young human oocytes, we found that MPS1 is required for cohesion protection (Figure 7E), but in contrast to mouse oocytes, there were similar requirements for SGO2 localization at both centromeres and pericentromeres (Figures 5C, 5D, 7C, and 7D). This precludes conclusions about the relevant pool of human SGO2 for cohesin protection. If the centromeric pool of SGO2 is indeed important for cohesion protection, as suggested by the mouse experiments, then an explanation is required for how this pool of SGO2-PP2A can access pericentromeric REC8 to dephosphorylate and thereby protect it. One possibility is that SGO2 initially localizes to centromere cups in metaphase I where it acquires its protection capability before relocalizing to the pericentromeric bridge where cohesin needs to be protected. Indeed, in cultured mitotic cells, centromeric Sgo1 bound to H2A-T120-P relocates to the pericentromere/inner centromere where it binds cohesin, aided by transcription.^51^ This translocation model can also explain how BUB1 and MPS1, which are localized at kinetochores, influence SGO2 localization at the pericentromeric bridge. To speculate, perhaps the role of MPS1 is to promote SGO2 binding to cohesin, explaining its critical requirement in cohesin protection in both mouse and human oocytes.

In our analysis of human oocytes, the predominant decline in SGO2 with age was observed at the pericentromeric bridge, although a modest reduction was observed at centromeres in metaphase I. As for pericentromeres (see above), the reduction in centromeric SGO2 with age could be caused by a reduction in cohesin at this location. An alternative explanation is that MPS1 and/or BUB1 function declines with age. However, BUB1 levels at kinetochores were comparable in younger and older human oocytes^7^ and MPS1 or BUB1 inhibition reduced SGO2 localization at both centromeres and pericentromeres, whereas age predominantly affected pericentromeric SGO2. We therefore favor the idea that cohesin loss is the key determinant leading to reduced SGO2 association with the pericentromeric bridge, resulting in an increased vulnerability of this highly specialized pool of cohesin.

### Age-dependent loss of cohesion protection as an aneuploidy driver

It is now well-established that erosion of cohesin is a major cause of age-related aneuploidy in human oocytes, with centromeric cohesion weakening being a key driver.^2,8,9^ Our study identifies the loss of SGO2-dependent protection as a further impediment to the safeguarding of centromeric cohesin with age.

## Star★Methods

Detailed methods are provided in the online version of this paper and include the following:

- KEY RESOURCES TABLE
- RESOURCE AVAILABILITY
  ○ Lead contact
  ○ Materials availability
  ○ Data and code availability
- EXPERIMENTAL MODEL AND STUDY PARTICIPANT DETAILS
  ○ Donation of human oocytes to research
- METHOD DETAILS
  ○ Generation of *eGFP-SGO2* mRNA for oocyte injection
  ○ *In vitro* transcribed mRNA synthesis
  ○ Immunofluorescence
  ○ Chromosome spreads
  ○ Human oocyte culture and treatments
  ○ Bovine oocyte collection
  ○ Super resolution imaging
  ○ Microinjection of Human and Bovine oocytes
  ○ Image processing and data analysis
- QUANTIFICATION AND STATISTICAL ANALYSIS

## Star★Methods

### Key Resources Table

**Table:** 

REAGENT or RESOURCE	SOURCE	IDENTIFIER
Antibodies		
human polyclonal anti-centromere protein antibody	Antibodies Incorporated, 15-235	RRID:AB_2939059
rabbit polyclonal anti-SGOL2 antibody	Novus Biologicals, NBP1-83567	NBP-83567 (no RRID)
mouse monoclonal anti-BUB1 antibody	Thermo Fisher Scientific Cat# MA1-5755	RRID:AB_2065922
mouse monoclonal anti-tubulin antibody	Sigma-Aldrich Cat# T6074	RRID:AB_477582
guinea pig polyclonal anti-CENP-C antibody	MBL International Cat# PD030	RRID:AB_10693556
mouse monoclonal anti-PP2A Catalytic alpha antibody	BD Biosciences Cat# 610556	RRID:AB_397910
mouse polyclonal anti REC8 antibody	Novus Cat# H00009985-B01P	RRID:AB_11014034
goat anti-rabbit Alexa Fluor 488 secondary antibody	ThermoFisher Scientific Cat# A-11008 (also A11008)	RRID:AB_143165
goat anti-mouse Alexa Fluor 555 secondary antibody	Thermo Fisher Scientific Cat# A-21422	RRID:AB_2535844
goat anti-human Alexa Fluor 647 secondary antibody	Thermo Fisher Scientific Cat# A-21445	RRID:AB_2535862
goat anti-guinea pig Alexa Fluor 647 secondary antibody	Thermo Fisher Scientific Cat# A-21450	RRID:AB_2535867
Biological samples		
198 human oocytes were donated by patients undergoing either IVF or ICSI treatment following informed consent and under HFEA license R0155. The project was approved by the NHS Research Ethics Committee (04/Q2802/26)	Edinburgh Fertility and Reproductive Endocrine Centre	Not applicable
Bovine oocytes were obtained after collection of ovaries from a local abattoir	Shotts, Lanarkshire	Not applicable
Chemicals, peptides, and recombinant proteins		
G-MOPS PLUS medium	Vitrolife	10130
G-IVF PLUS	Vitrolife	10136
Mineral oil	Merck	8042-47-5
Reversine	Cayman Chemical Research	10004412
BAY-320	MedChemExpress	HY-104000
HEPES buffered M199	Gibco BRL, Life Technologies Ltd.	12340030
sodium pyruvate	Merck	S8636
L-glutamine	ThermoFisher Scientific	A2916801
bovine serum albumin	Merck	A4503
Penicillin-Streptomycin	Merck	P4333
Leibovitz’s L-15 Medium	ThermoFisher Scientific	11415064
BO-IVM medium	IVF Biosciences	71001
ProLong Gold hard-set antifade mountant with Hoechst	ThermoFisher Scientific	P36931
Tyrode’s Solution, Acidic	Merck	T1788
SlowFade Glass Soft-set antifade mountant	ThermoFisher Scientific	S36917
Critical commercial assays		
mMESSAGE mMACHINE™ T7 Transcription Kit	Ambion	AM1344
RNeasy Mini Kit	QIAGEN	74004
Oligonucleotides		
gtaggtacagcagatgtgactATGGAGTGCCCAGTGATGGAA	Merck – custom synthesised	AMo_11079
gtgacactatagaatagggccctctagaTTATCTTCTCA TCTTGTCTCTGAGGCTTG	Merck – custom synthesised	AMo_11080
ggaATGGTGAGCAAGGGCGAGGA	Merck – custom synthesised	AMo_10982
agtcacatctgctgtacctacCTTGTACAGCTCGTCCATGCCG	Merck – custom synthesised	AMo_10983
TAATCTAGAGGGCCCTATTCTATAGTGTCA CCTAAATGCTAGAGC	Merck – custom synthesised	AMo_11282
TCCTCGCCCTTGCTCACCATtccggtgGCGGTA CCAAGCTTGGGTCTC	Merck – custom synthesised	AMo_11283
Recombinant DNA		
pcDNA3 mRNA expression vector	Invitrogen	V79020
H2B-mCherry expression vector	Addgene Cat#20972	RRID:Addgene_20972
hSGO2-eGFP expression vector	This paper	AMp2188
eGFP-hSGO2 expression vector	This paper	AMp2329
Software and algorithms		
COIL-Edinburgh/SPB_Distances	This paper	https://zenodo.org/doi/10.5281/zenodo.10026234
Zeiss Zen 3.3 (blue edition)	Zeiss	https://www.zeiss.com/microscopy/en/products/software/zeiss-zen.html
Fiji	National Institutes of Health	https://fiji.sc/
SyGlass	SyGlass	https://www.syglass.io/
iMaris	Oxford Instruments	https://imaris.oxinst.com/
Prism 9	Graphpad	https://www.graphpad.com/features
R studio	R	https://posit.co/download/rstudio-desktop/
Adobe Illustrator	Adobe	https://www.adobe.com/uk/

## Resource Availability

### Lead contact

Further information and requests for resources and reagents should be directed to and will be fulfilled by the lead contact, Adele Marston (adele.marston@ed.ac.uk).

### Materials availability

Plasmids generated in this study are available without restriction from the lead contact.

## Experimental Model and Study Participant Details

### Donation of human oocytes to research

The NHS Research Ethics Committee approved the research project (Indicators of Oocyte and Embryo Development, 04/Q2802/26) and all work was conducted under a Research Licence from the Human Fertilisation and Embryology Authority (HFEA; R0155; Indicators of Oocyte and Embryo Development). Informed consent for donation of oocytes and embryos to research was provided by couples undergoing intracytoplasmic sperm injection (ICSI) or in vitro fertilisation (IVF) at the Edinburgh Fertility and Reproductive Endocrine Centre or the Centre for Reproductive Medicine (CRM), University Hospitals Coventry and Warwickshire (UHCW) NHS Trust. Donations were optional and did not affect the treatment received. Couples were aware of the purpose of the research and were not provided compensation. Material donated to research only included oocytes that could not be used for treatment and would have otherwise been disposed of including immature oocytes and inseminated but unfertilised oocytes. Ovarian stimulation was induced using FSH according to standard clinical protocols, either GnRH agonist or antagonist regimens. Oocytes were cultured in the Vitrolife media suite. After clinical embryologists had completed ICSI or IVF procedures, research material was identified and made available for collection by licensed researchers. Immature oocytes arising from ICSI treatments were collected from the clinic 4-6 hours after oocyte retrieval. GV oocytes were checked every half hour under the microscope for GVBD. If they did not undergo GVBD within 10 hours after removal from the ovary, they were not used in the experiment. Unfertilised or misfertilised metaphase II oocytes from ICSI or IVF were collected from the clinic 3-5 hours after the fertilisation check had been completed by a clinical embryologist ∼22 hours after insemination. Cells were transported in G-MOPS PLUS medium (Vitrolife, #10130) at 37°C in a portable incubator (K Systems) for approximately 15 min to the research laboratory. Cells were then cultured in G-IVF PLUS (Vitrolife, #10136) under mineral oil (Merck, # 8042-47-5) at 37°C in 5% O_2_, 6% CO_2_ and 89% N_2_. No oocytes were vitrified or thawed in this study.

A total of 198 oocytes from 120 donors, from November 2019 to October 2023, were used in this study.

## Method Details

### Generation of *eGFP-SGO2* mRNA for oocyte injection

The human *SGO2* coding sequence was custom synthesised (Invitrogen GeneArt) and inserted into a pcDNA3 mRNA expression vector (Invitrogen) in frame with a 5’ eGFP coding sequence to generate plasmid AMp2329 (pDNA3-eGFP-hSGO2). The *SGO2* coding sequence was amplified from AMp2188 (with primers 11079 (gtaggtacagcagatgtgactATGGAGTGCCCAGTGATGGAA) and 11080 (gtgacactatagaatagggccctctagaTTATCTTCTCATCTTGTCTCTGAGGCTTG), the eGFP coding sequence was amplified with primers 10982 (ggaATGGTGAGCAAGGGCGAGGA) and 10983 (agtcacatctgctgtacctacCTTGTACAGCTCGTCCATGCCG), the pcDNA3 backbone was amplified with primers 11282 (TAATCTAGAGGGCCCTATTCTATAGTGTCACCTAAATGCTAGAGC) and 11283 (TCCTCGCCCTTGCTCACCATtccggtgGCGGTACCAAGCTTGGGTCTC). The PCR fragments were fused together by Gibson assembly.^52^

### *In vitro* transcribed mRNA synthesis

Plasmids containing the coding sequence of human SGO2 and H2B-mCherry were obtained from AMp2329 (see above) and Addgene, #20972,^53^ respectively. H2B-mCherry was linearized by DraIII (New England Biolabs, R3510S). After linearization, the digests were purified using Promega PCR Purification kit (Wizard® SV Gel and PCR Clean-Up System, Promega, A9281). Synthesis of cRNAs was performed using T7 polymerase (mMESSAGE mMACHINE™ T7 Transcription Kit, Ambion, AM1344) according to the manufacturer’s instructions. The mRNA was purified using a RNeasy kit (RNeasy Mini Kit, QIAGEN, 74004).

### Immunofluorescence

Whole oocytes were fixed and immunostained as previously described with minor modification.^9^ Briefly, cells were washed through warmed PHEM buffer (60 mM PIPES, 25 mM HEPES, 10 mM EGTA, 4 mM MgSO_4_.7H_2_O; pH 6.9) with 0.25% Triton X-100 at 37°C. Cells were then fixed with 4% formaldehyde in PHEM buffer with 0.25% Triton X-100 for 30 min and permeabilised further in PBS with 0.25% Triton X-100 for 15 min at room temperature. Cells were then stored in PBS with 0.05% Tween-20 (PBST) at 4°C for up to two weeks until they were immunostained. For immunofluorescence, oocytes were blocked in 3% BSA in PBST at 4°C overnight before being incubated in primary antibodies. Cells were incubated in human polyclonal anti-centromere protein antibody (1:50; Antibodies Incorporated, 15-235), rabbit polyclonal anti-SGOL2 antibody (1:200; Novus Biologicals, NBP1-83567), mouse monoclonal anti-BUB1 antibody (1:100; ThermoFisher Scientific, MA15755), mouse monoclonal anti-tubulin antibody (1:200; T6074, Sigma) and guinea pig polyclonal anti-CENP-C antibody (1:200; MBL, PB030) 3% BSA in PBST at 4°C overnight. Following 3 × 20 min washes in 1% BSA in PBST, cells were incubated in goat anti-rabbit Alexa Fluor 488 (1:500; A-11008, ThermoFisher Scientific), goat anti-mouse Alexa Fluor 555 secondary antibody (1:500; A-21422, ThermoFisher Scientific), goat anti-human Alexa Fluor 647 (1:500; A-21445, ThermoFisher Scientific), and goat anti-guinea pig Alexa Fluor 647 secondary antibody (1:500; A-21450, ThermoFisher Scientific) in 3% BSA in PBST for 1 hr at room temperature and then washed 3 x 20 min in 1% BSA in PBST. Oocytes were mounted in ProLong Gold hard-set antifade mountant with Hoechst (P36931, ThermoFisher Scientific) mixed 1:1 with PBS on a FluoroDish (FD35-100, WPI) and incubated in the dark overnight at room temperature to allow the mounting medium to harden. Cells were stored in the dark at 4°C and imaged within two weeks of being mounted.

### Chromosome spreads

Chromosome spreads of human oocytes were prepared by adapting a previously described method for mouse oocytes.^54^ Briefly, oocytes were incubated in Tyrode’s Solution, Acidic (T1788, Merck) at 37°C for approximately 20-30 seconds until the zona pellucida dissolved. The oocytes were then washed through three wells containing G-IVF PLUS (Vitrolife #10136) at 37°C. Oocytes were then dropped into a well of a 12 well slide (Epredia, X1XER302W) containing 30μl of fixing solution (3mM DTT, 1% formaldehyde and 0.15% Triton-X-100; PH 9.2-9.3) and allowed to burst. Slides were slowly air dried in slightly open humidified chamber overnight at room temperature. Chromosome spreads were immediately used for immunostaining or frozen at -20°C and used within two weeks. For immunostaining, chromosome spreads were washed for 3 x 5 min in PBST before being incubated in the primary antibodies. Spreads were incubated in mouse monoclonal anti-PP2A Catalytic α antibody (1:50; BD Biosciences, 610556), rabbit polyclonal anti-SGOL2 antibody (1:200; Novus Biologicals, NBP1-83567), mouse polyclonal anti REC8 antibody (1:50; Novus Biologicals, H00009985-B01P) human anti-CREST antibody/antisera (1:50 Antibodies Incorporated, 15-235) and guinea pig polyclonal antibody anti-CENP-C antibody (1:200; MBL, PB030) diluted in 3% BSA, 7% FBS in PBST. Chromosome spreads were then washed through 1% BSA in PBST and incubated in goat anti-rabbit Alexa Fluor 488 (1:500; A-11008, ThermoFisher Scientific), goat anti-mouse Alexa Fluor 555 secondary antibody (1:500; A-21422, ThermoFisher Scientific) and goat anti-guinea pig Alexa Fluor 647 secondary antibody (1:500; A-21450, ThermoFisher Scientific) in 3% BSA in PBST for 1 hr at room temperature. Chromosome spreads were counterstained with Hoechst 33258 (20 μg/ml) diluted in PBST for 5 min at room temperature and were then mounted using SlowFade Glass Soft-set antifade mountant (ThermoFisher Scientific, S36917) and 1.5H coverslips (VWR, 631-0121). Slides were then sealed with clear nail polish (Revlon) and incubated in the dark overnight at room temperature to allow the mounting medium to set. Spreads were stored in the dark at 4°C and imaged within two weeks of being mounted.

### Human oocyte culture and treatments

Oocytes were cultured in G-IVF PLUS (Vitrolife, #10136) under mineral oil (Merck, # 8042-47-5) at 37°C in 5% O_2_, 6% CO_2_ and 89% N_2_. The stage of oocyte meiosis was visually determined as Prophase I by the presence of a germinal vesicle nucleus (GV) and Metaphase II by the presence of a polar body. Oocytes were matured to prometaphase I, metaphase I and telophase I 10, 12, and 14 hours after nuclear envelope breakdown.^28,55^ Oocyte stages were verified by chromosome conformation and tubulin immunolocalisation.

For MPS1 and BUB1 inhibition at metaphase II, oocytes were treated with 500nM reversine (Cayman Chemical Research, 10004412)^28^ or 10μM BAY-320 (MedChemExpress, HY-104000),^47,56^ respectively for 16 hrs prior to fixation, or with an appropriate volume of DMSO as a control. To inhibit MPS1 from prophase I, oocytes were allowed to undergo NEBD and were then incubated with 500nM reversine or 5μM BAY-320, for 20 hours to allow maturation to metaphase II.

### Bovine oocyte collection

Bovine ovaries were collected from a local abattoir (Shotts, Lanarkshire) and transported to the laboratory in pre-warmed HEPES buffered M199 (Gibco BRL, Life Technologies Ltd., Paisley, Renfrewshire, UK) medium supplemented with sodium pyruvate (2 mM), glutamine (2 mM), bovine serum albumin (BSA) (3 mg/ml), penicillin G (75 μg/ml), streptomycin (50 μg/ml): all chemicals were from Merck Life Sciences UK, Gillingham, Poole, Dorset, UK). On arrival at the laboratory (within one hour), ovaries were washed three times in sterilized phosphate-buffered saline. Cumulus oocyte complexes (COCs) were aspirated from medium-sized follicles (3–6 mm in diameter) using a 10-ml disposable syringe connected to an 18-gauge needle. Then COCs with multi-layers of surrounding cumulus cells and oocytes with homogeneous cytoplasm were selected. Denuded, small oocytes and those with heterogenous cytoplasm were discarded. Searching and selection of COCs was done under a stereomicroscope equipped with heating stage in pre-warmed Leibovitz’s L-15 Medium (ThermoFisher Scientific) supplemented with Bovine serum albumin (BSA, 0.3% W/V), (2% sodium pyruvate, V/V), (1%, L-glutamine, V/V), penicillin G (75 μg/ml) and streptomycin (50 μg/ml). The collected COCs were cleaned from the surrounded cumulus cells by vortex for 3 minutes in L-15 medium. Oocytes were matured in BO-IVM medium (IVF Biosciences, Falmouth, Cornwall, UK) at 38° C, 7% CO_2_ for 18-24 hours.

### Super resolution imaging

Fixed cells and spreads were imaged using LSM880 or LSM980 laser scanning confocal equipped with an Airyscan 1 or Airyscan 2 detector, respectively (Zeiss UK, Cambridge), using a Plan-APO (63x/1.4 NA) oil objective (Zeiss). 0.14- 0.25 μm optical section spacing was used to encompass entire chromatin structures. 405nm, 488nm, 561nm and 639nm lasers were used to detect DAPI and Hoechst staining and Alexa Fluor 488 Alexa Fluor 555 and Alexa Fluor 647, respectively. For live imaging, 2- 2.5 μm optical section spacing was used to encompass entire oocyte. 488nm, 561nm and 639nm lasers were used to detect exogenous eGFP-SGO2, H2B-mCherry and sir-DNA labelled chromosomes, respectively. Emission filters were used to prevent emissions from neighbouring wavelength. Material used for signal intensity measurements were imaged using the same parameters. Airyscan images were subject to 3D Airyscan Processing using the Auto Filter and Medium Strength functions using Zeiss Zen 3.3 (blue edition) software for pixel reassignment and deconvolution to generate 120 nm resolution images. Representative images were prepared using Fiji software (National Institutes of Health).

### Microinjection of Human and Bovine oocytes

Human prophase I (GV) oocytes, collected from ICSI patients, were handled in G-MOPS medium. Using the same medium under oil, the oocytes were microinjected with human *eGFP-SGO2 in vitro* transcribed mRNA in a concentration of 150 ng/μl. After microinjection, oocytes were transferred into G-IVF medium containing the DNA labelling dye, sir-DNA (#CY-SC007, Cytoskeleton, 500 nM) for at least two hours before imaging.

The isolated COCs from bovine ovaries were handled in Leibovitz’s L-15 Medium, then cumulus cells were removed by vortex for 3 minutes. These denuded oocytes were microinjected with human eGFP-SGO2 (400ng/μl) and H2B mCherry (250 ng/μl) *in vitro* transcribed mRNAs in L-15 Medium under oil. After microinjection, the oocytes were kept in L-15 medium at 38°C for at least two hours to allow RNA translation. Then, oocytes were transferred to BO-IVM medium before imaging. Human and bovine oocytes were micro-injected using an Eppendorf FemtoJet 4i system equipped with a heated stage. Oocytes were imaged every 20 minutes in a condition of 7%CO_2_, at 37°C for human oocytes or 38°C for bovine oocytes using a Zeiss LSM 980 Airyscan microscope with 488nm and 439nm lasers and 2μm optical sections, encompassing the whole oocyte.

### Image processing and data analysis

Image processing and quantification were performed with Fiji software. Integrated density (sum density) was used to measure fluorescence intensity of selected regions. This represents the mean grey value relative to the selected area. The free hand selection tool was used to define the region surrounding the kinetochore as shown in the examples with dotted lines in Figures 1–3 and 5–7; Figure S1. The integrated density of the kinetochore/centromeric SGO2 was normalised to the integrated density of the kinetochore it surrounded. The background fluorescence was measured at four locations on the image and averaged. For determination of fluorescence intensity, the normalized fluorescence (NF), was used as described in the following equation: NF = integrated fluorescence intensity – (area × average background fluorescence).

To measure inter kinetochore distance, kinetochores from sister chromatids were manually identified and distance measurements were taken from their peak intensities. Inter kinetochore distances were measured in 3D using a custom macro (https://zenodo.org/doi/10.5281/zenodo.10026234). All measurement analysis included between 80-100% of available centromere intensities and kinetochores pairs to minimise bias. Chromosomes measured were clearly paired. Ambiguous pairs were excluded from analysis. SGO2, PP2A and REC8 between sister chromatids were scored as joined “bridge” or separated – “no bridge” manually using Fiji based on an absence of signal between kinetochores, relative to the background. Single chromatids were counted manually in Fiji and were confirmed using SyGlass virtual reality software.

The meiosis I chromosome spreads in Figure S4 were analysed by using the free-hand tool to define the region of the kinetochore using the CENP-C marker (used for centromeric signal of SGO2 and PP2A and for CENP-C kinetochore signal) and the total SGO2 and PP2A signal in SUM projections of Z-stacks. Non-centromeric signal was calculated by subtracting the centromeric SGO2 or PP2A CENP-C normalised signal from the respective total SGO2 or PP2A CENP-C normalised signal.

Live imaging files were also subjected to 3D Airyscan Processing using the Auto Filter and Medium Strength functions using Zeiss Zen 3.3 (blue edition) then it converted into iMaris compatible files to merge the maximum projections and generate the video files (imaris 9 software). Imaris was also used to create representative micrographs of indicated timepoints.

## Quantification and Statistical Analysis

Statistical analysis and graphs were generated using Graphpad Prism 9 software (San Diego), except for the plots and analysis in Figures S4E and S4F, for which the R software was used. Micrographs and graphs were assembled using Adobe Illustrator. Statistical tests were performed using Mann-Whitney test or Kruskal-Wallis test for non-paired and non-normally distributed data. Chi square test was used to test the association between categorical variables in Figure 4. Linear regression and sigmoidal, 4 parameter logistic fit were selected based on best fit (R=1), as detailed in the figure legends. P values are designated as *P < 0.05, **P < 0.01, ***P < 0.001, and ****P < 0.0001. Non-significant values are indicated as n.s. The number of replicates is indicated in the figure legends. All violin plots show median (dashed black line), 25th and 75th percentiles (dotted black lines). Statistical details of all experiments are given in the figure legends.

## Supplementary Material

Supplemental information can be found online at https://doi.org/10.1016/j.cub.2023.11.061.

Extended view format

Supplemental information

Video S1

Video S2

## Data Availability

All data reported in this paper will be shared by the lead contact upon request. All original code is deposited at zenodo (https://zenodo.org/doi/10.5281/zenodo.10026234) and is publicly available as of the date of publication. DOIs are listed in the key resources table. Any additional information required to reanalyze the data reported in this paper is available from the lead contact upon request.

## Highlights

- SGO2 localizes to centromeric cups and the pericentromeric bridge in human oocytes
- Human SGO2 prevents premature loss of cohesion and aneuploidy during meiosis
- Bridge SGO2 is lost in human oocytes from women of advanced age
- MPS1 and BUB1 kinases contribute to SGO2 localization in human oocytes

## In brief

Human fertility declines with maternal age due to aneuploidy affecting the oocyte. Mihalas et al. use super-resolution microscopy in human oocytes and provide evidence that age-dependent loss of the SGO2 cohesin protector protein could contribute to this phenomenon.
