# Supplementary material for: Age-dependent loss of cohesion protection in human oocytes
Source: Curr Biol. Author manuscript; Available in PMC 2025 May 9. (PMC7617652; doi:10.1016/j.cub.2023.11.061)
Supplement: Supplemental information [file EMS204561-supplement-Supplemental_information.pdf]

Current Biology, Volume *34*

## Supplemental Information

# Age-dependent loss of cohesion protection in human oocytes

**Bettina P. Mihalas, Gerard H. Pieper, Mansour Aboelenain, Lucy Munro, Vlastimil Srsen, Cerys E. Currie, David A. Kelly, Geraldine M. Hartshorne, Evelyn E. Telfer, Andrew D. McAinsh, Richard A. Anderson, and Adele L. Marston**

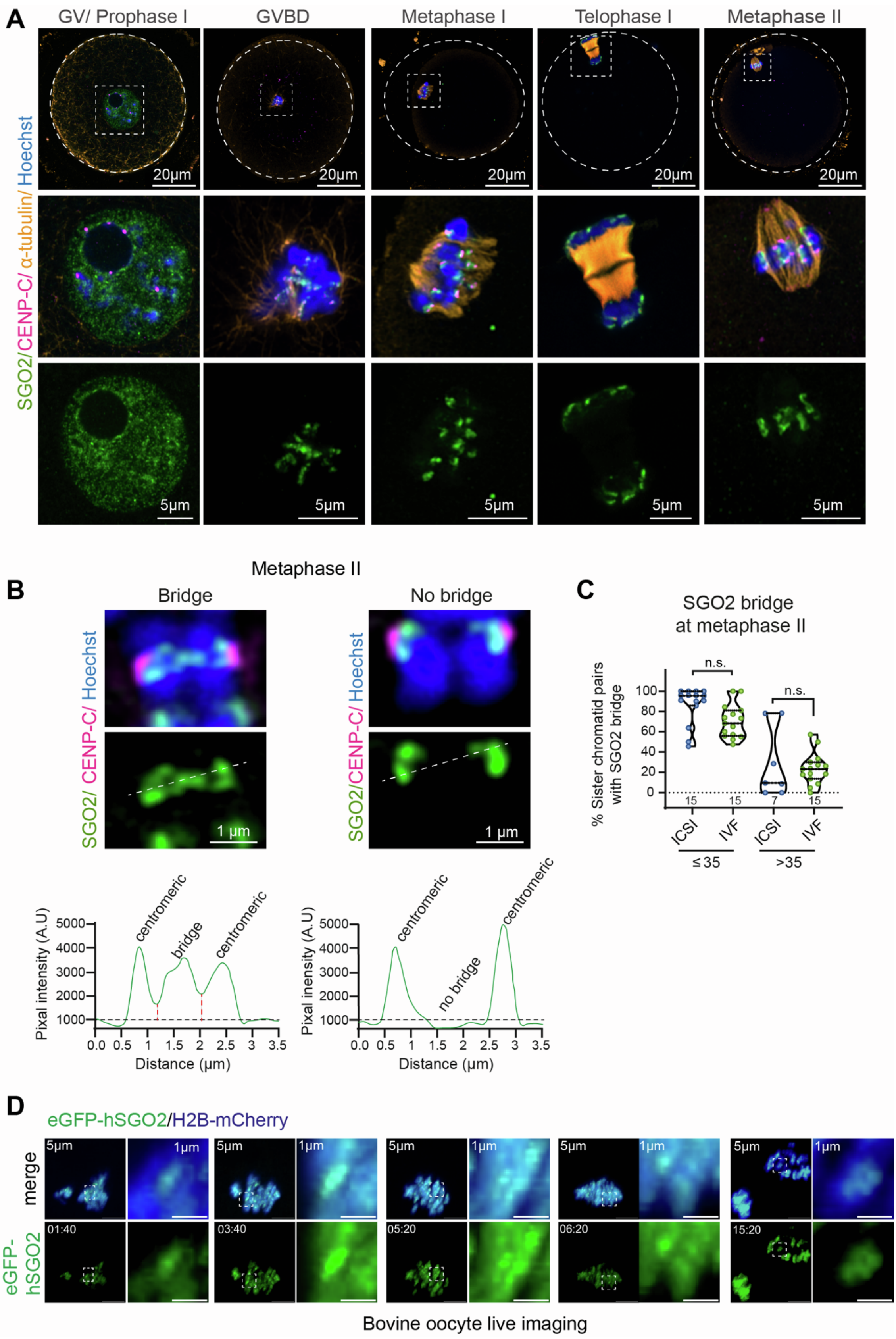


**Figure S1. SGO2 localisation throughout meiosis. Related to Figure 1**

(A) SGO2 localization in GV, GVBD, metaphase I, telophase I, and metaphase II stage oocytes. Oocytes were immunostained with antibodies against SGO2 (green), CENP-C (inner kinetochores, magenta), $\alpha$-tubulin (microtubules, orange) and counter stained with Hoechst (blue). White circles with dashed lines represent the oocyte circumference. White boxes with dashed lines represent chromosome masses that have been further magnified below. (B) Representative line scans showing distinction between bridge and centromeric SGO2 pools. A line was drawn as shown on images of individual sister chromatid pairs from metaphase II oocytes and the fluorescence profile of SGO2 is shown in the graph. (C) Comparison of SGO2 localization at the pericentromeric bridge in metaphase II oocytes from women stratified by age (≤35 years or >35 years) undergoing ICSI or IVF treatment. Plots show median (dashed black line), 25th and 75th percentiles (dotted black lines). n.s. = not significant (Kruskal-Wallis test). (D) Live imaging showing the localization of the exogenous hSGO2 in bovine oocytes. Representative image showing eGFP-hSGO2 (green), H2B-mCherry (blue) at different time points (hh:mm) of meiosis I. White dashed line boxes indicate DNA and eGFP-hSGO2 further magnified in bottom panels. See also Videos S1 and S2.

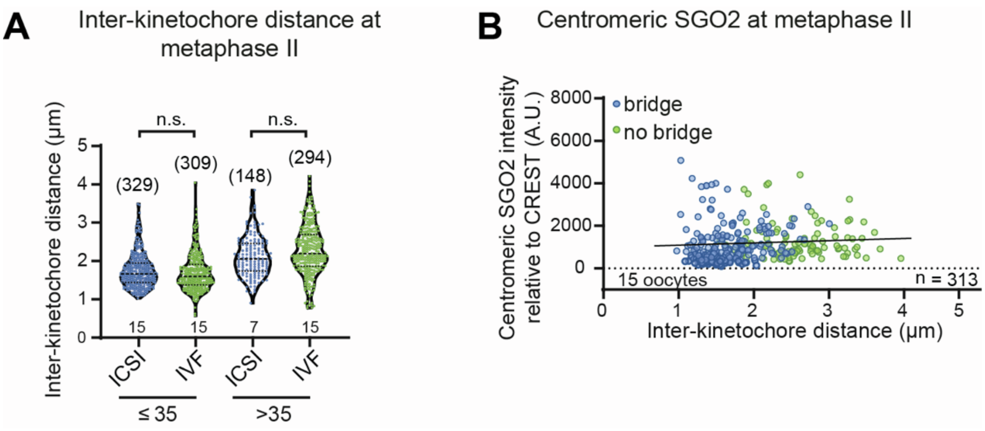


**Figure S2. Treatment regime (IVF or ICSI) does not affect age-dependent cohesion loss in metaphase II oocytes and the centromeric pool of SGO2 does not correlate with inter-sister kinetochore distance. Related to Figure 2.**

(A) Comparison of inter-kinetochore distance at metaphase II oocytes from women aged ≤35 or >35 years separated into those undergoing ICSI or IVF treatment. Plots show median (dashed black line), 25th and 75th percentiles (dotted black lines). n.s. = not significant (Kruskal-Wallis test). (B) Centromeric SGO2 does not correlate with inter-sister kinetochore distance in metaphase II. The relative intensity of the centromeric pool of SGO2 metaphase II oocytes was measured in arbitrary units (A.U.) relative to the kinetochore marker CREST ($P$ = 0.052; $R^2$ = 0.012). Data were fit to a linear regression.

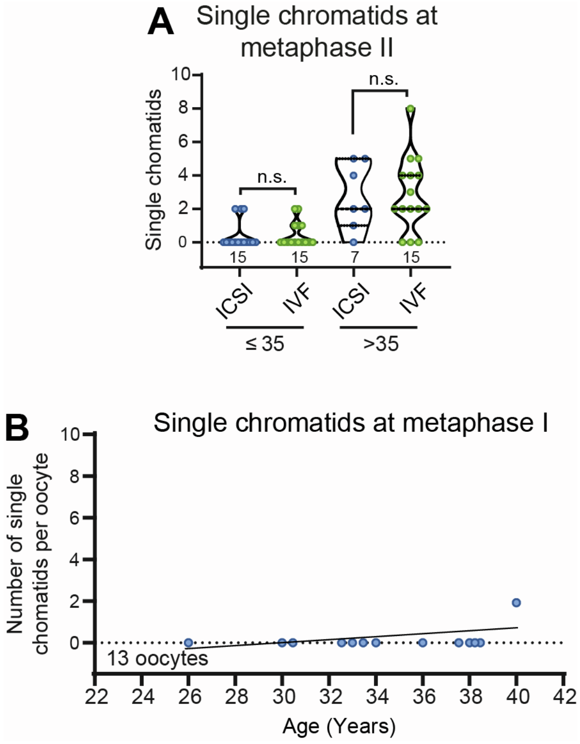


**Figure S3. Increased frequency of single chromatids with age is observed only in metaphase II. Related to Figure 3.**

(A) The number of single chromatids in metaphase I oocytes was scored relative to woman's age. Metaphase I oocytes were stained as in Figure 1 (Kruskal-Wallis test). Plots show median (dashed black line), 25th and 75th percentiles (dotted black lines). *P* values were calculated using the Mann-Whitney test. n.s., Not significant. (B) The number of single chromatids at metaphase I were scored relative to woman's age (≤35 and >35). Data were fit to a linear regression ($R^2$ = 0.1699; *P* = 0.1617).

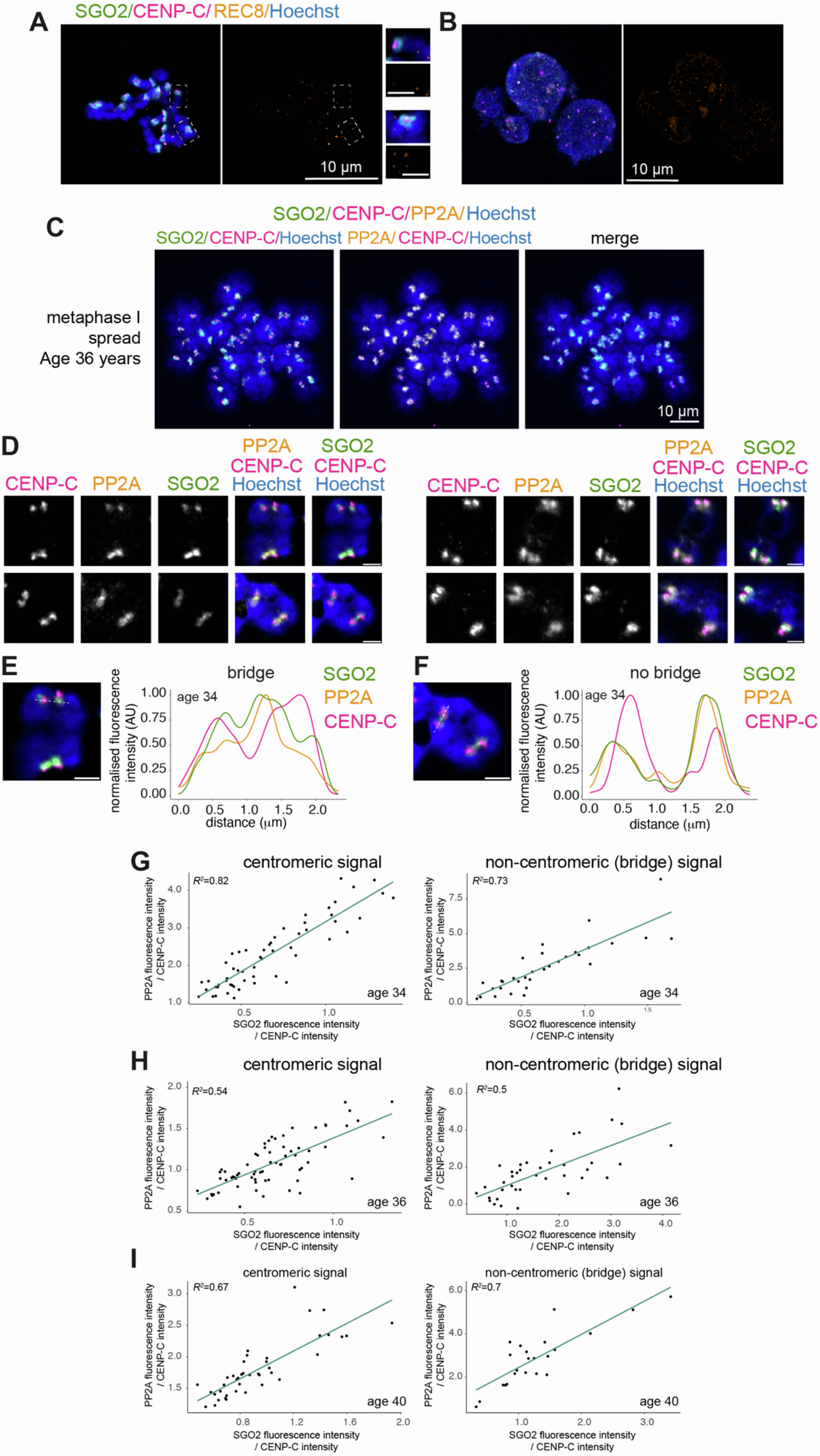

A
SGO2/CENP-C/REC8/Hoechst
10 µm
B
10 µm
C
SGO2/CENP-C/PP2A/Hoechst
SGO2/CENP-C/Hoechst
PP2A/CENP-C/Hoechst
merge
metaphase I spread Age 36 years
10 µm
D
CENP-C
PP2A
SGO2
PP2A CENP-C Hoechst
SGO2 CENP-C Hoechst
E
bridge
age 34
SGO2
PP2A
CENP-C
normalised fluorescence intensity (AU)
distance (µm)
F
no bridge
age 34
G
centromeric signal
non-centromeric (bridge) signal
R²=0.82
R²=0.73
age 34
PP2A fluorescence intensity / CENP-C intensity
SGO2 fluorescence intensity / CENP-C intensity
H
R²=0.54
R²=0.5
age 36
I
R²=0.67
R²=0.7
age 40

**Figure S4. SGO2 and PP2A co-localize at centromere cups and pericentromere bridges in metaphase I. Related to Figure 4.**

(A and B) The REC8 antibody is specific. REC8 signal was not detected in pronuclei of the zygote (A) or on decondensed sperm (B) using immunofluorescence and imaging conditions as in Figure 4C and D. Spread metaphase I human oocytes stained with antibodies against SGO2, PP2A, CENP-C and counter-stained with DAPI. Examples of a full (C) metaphase I spread and individual bivalents (D) from different spreads are shown. (E and F) Line scans of example bivalents where a bridge structure is present (E) or absent (F). (G-I) Correlation of PP2A and SGO2 signal intensity at individual centromeres and non-centromeres (pericentromeric bridge) for 3 human oocytes are shown. Analysis in G and H is from the same metaphase I spread shown in E and F.

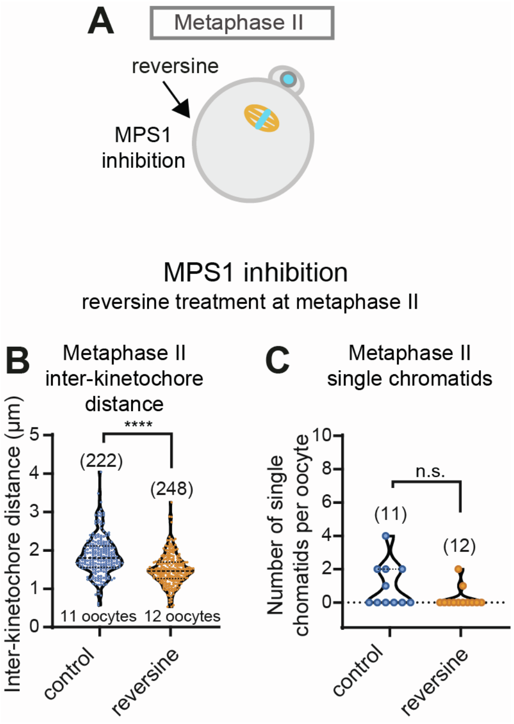


**Figure S5. Effect of MPS1 inhibition on inter-kinetochore distances and single chromatids. Related to Figure 5.**

(A) Scheme of experiment. (B) Inter-kinetochore distance and the (C) the number of single chromatids identified in control and metaphase II oocytes from women aged ≤ 36 years treated with reversine as in Figure 5. Plots show median (dashed black line), 25th and 75th percentiles (dotted black lines). ****P <0.0001 (Mann-Whitney test). n.s., Not significant (P=0.12, Mann-Whitney test).

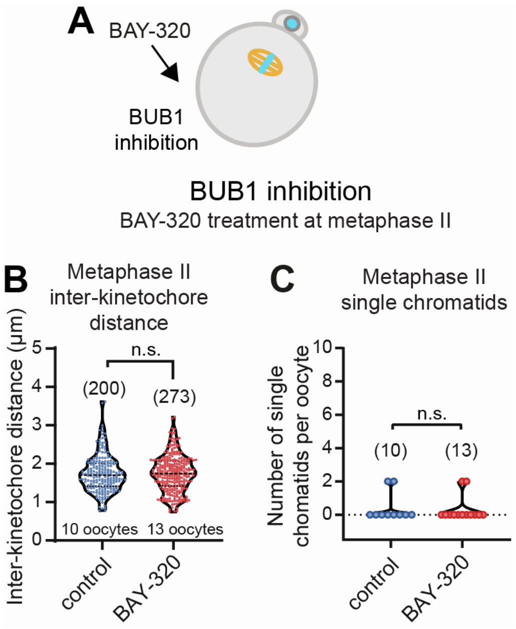


**Figure S6. Effect of BUB1 inhibition on inter-kinetochore distances and single chromatids. Related to Figure 6.**

(A) Scheme of the experiment from Figure 6A. (B) Inter-kinetochore distance and (C) the number of single chromatids identified in control and metaphase II oocytes from women aged ≤ 36 years treated with BAY-320 as in Figure 5. Plots show median (dashed black line), 25th and 75th percentiles (dotted black lines). n.s. Not significant; Mann-Whitney test (For (B) *P* >0.56; (C) P>0.9999).

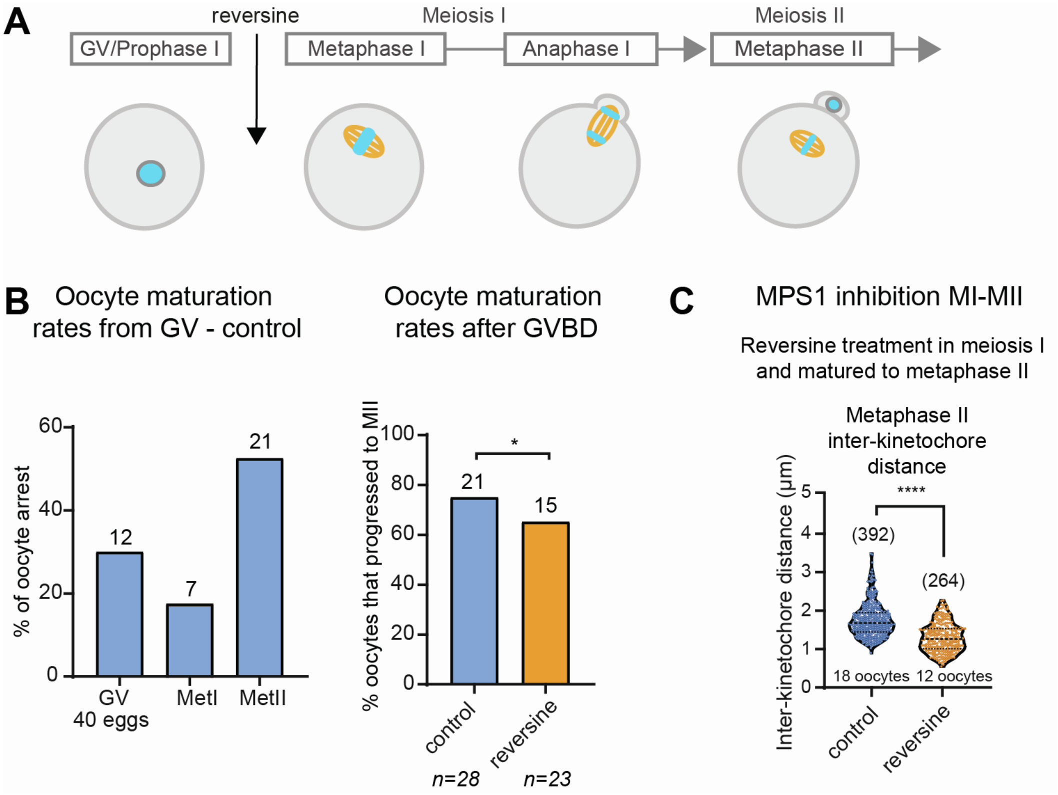


**Figure S7. Effect of MPS1 inhibition at GVBD on inter-sister kinetochore distance at metaphase II, Related to Figure 7.**

(A) Schematic of the experiment reproduced from Figure 7A. (B) Rates of oocyte maturation *in vitro.* GV oocytes were cultured *in vitro* and inspected for GVBD every 30 min. If GVBD did not occur with 10h after removal from the ovary, they were scored as GV arrest (19% overall: 12 oocytes out of a total of 63 combining DMSO and reversine-treated). In total, 28 oocytes undergoing GVBD were treated with DMSO, of which 21 progressed to metaphase II. Out of 23 oocytes undergoing GVBD and treated with reversine, 15 progressed to metaphase II. Statistical analyses were performed using the Chi-squared test (*$P$ <0.0089). (C) Decrease in inter-sister kinetochore distance in oocytes treated with reversine at GVBD and matured to metaphase II as in Figure 7A-D (****P<0.0001, Mann-Whitney test).
